# ROS regulation of RAS and vulva development in *Caenorhabditis elegans*

**DOI:** 10.1371/journal.pgen.1008838

**Published:** 2020-06-16

**Authors:** Maximilian Kramer-Drauberg, Ju-Ling Liu, David Desjardins, Ying Wang, Robyn Branicky, Siegfried Hekimi

**Affiliations:** Department of Biology, McGill University, Montreal, Quebec, Canada; University of California San Francisco, UNITED STATES

## Abstract

Reactive oxygen species (ROS) are signalling molecules whose study in intact organisms has been hampered by their potential toxicity. This has prevented a full understanding of their role in organismal processes such as development, aging and disease. In *Caenorhabditis elegans*, the development of the vulva is regulated by a signalling cascade that includes LET-60ras (homologue of mammalian Ras), MPK-1 (ERK1/2) and LIN-1 (an ETS transcription factor). We show that both mitochondrial and cytoplasmic ROS act on a gain-of-function (gf) mutant of the LET-60ras protein through a redox-sensitive cysteine (C118) previously identified in mammals. We show that the prooxidant paraquat as well as *isp-1*, *nuo-6* and *sod-2* mutants, which increase mitochondrial ROS, inhibit the activity of LET-60rasgf on vulval development. In contrast, the antioxidant NAC and loss of *sod-1*, both of which decrease cytoplasmic H_2_0_2_, enhance the activity of LET-60rasgf. CRISPR replacement of C118 with a non-oxidizable serine (C118S) stimulates LET-60rasgf activity, whereas replacement of C118 with aspartate (C118D), which mimics a strongly oxidised cysteine, inhibits LET-60rasgf. These data strongly suggest that C118 is oxidized by cytoplasmic H_2_0_2_ generated from dismutation of mitochondrial and/or cytoplasmic superoxide, and that this oxidation inhibits LET-60ras. This contrasts with results in cultured mammalian cells where it is mostly nitric oxide, which is not found in worms, that oxidizes C118 and activates Ras. Interestingly, PQ, NAC and the C118S mutation do not act on the phosphorylation of MPK-1, suggesting that oxidation of LET-60ras acts on an as yet uncharacterized MPK-1-independent pathway. We also show that elevated cytoplasmic superoxide promotes vulva formation independently of C118 of LET-60ras and downstream of LIN-1. Finally, we uncover a role for the NADPH oxidases (BLI-3 and DUOX-2) and their redox-sensitive activator CED-10rac in stimulating vulva development. Thus, there are at least three genetically separable pathways by which ROS regulates vulval development.

## Author summary

Reactive oxygen species (ROS) are signalling molecules whose study in intact organisms has been hampered by their potential toxicity. In *Caenorhabditis elegans*, the development of the vulva is regulated by a signal transduction cascade that includes LET-60ras (the worm homologue of mammalian RAS), MPK-1 (the worm homologue of ERK1/2), and LIN-1 (an ETS transcription factor). Here, using *C*. *elegans* vulva development as a model, we show that both mitochondrial and cytoplasmic ROS act on the worm homologue of RAS (LET-60ras) through a redox-sensitive cysteine (C118). However, in contrast to what is observed in cultured mammalian cells, we find that C118 is oxidized by hydrogen peroxide, rather than by superoxide or nitric oxide, and that its oxidation inhibits rather than activates the pathway. Furthermore, we find that the regulation of LET-60ras by oxidation does not act through MPK-1 to affect vulva development. We also identify two additional ROS signalling pathways that promote vulva development. Elevated cytoplasmic superoxide promotes vulva formation independently of C118 of LET-60ras and downstream of LIN-1, whereas the NADPH oxidases (BLI-3 and DUOX-2) and their redox-sensitive activator CED-10rac act in yet another parallel pathway to promote vulval formation.

## Introduction

Reactive oxygen species (ROS) are signalling molecules that participate in regulating many cellular processes [1–3], including in *C*. *elegans* [4–8]. Superoxide (O_2_^•-^) is produced by a variety of processes, including intracellularly by mitochondrial respiration [9], enzymes such as cytochrome P450 and aldehyde oxidase [10, 11], as well as extracellularly by membrane-bound NAPDH oxidases (NOXs) [12]. Hydrogen peroxide (H_2_O_2_) is produced by the action of superoxide dismutases, which convert superoxide to hydrogen peroxide [13], as well as directly by enzymes like xanthine oxidase [14] and monoamine oxidase [15]. It is also produced by the Dual Oxidases (DUOXs), a sub-class of NOX proteins that possess a peroxidase domain, and can thus convert superoxide to hydrogen peroxide [16]. Several distinct enzymatic systems, such as the Catalases (Ctls) [17], Glutathione peroxidases (Gpxs) [18], Thioredoxins (Trxs) and Peroxiredoxins (Prdxs) [19] remove hydrogen peroxide, thus terminating any signal it might carry. In *C*. *elegans*, there are five distinct superoxide dismutases (SODs), with SOD-2 the main mitochondrial matrix enzyme, SOD-1 the main cytoplasmic and mitochondrial inter-membrane space enzyme, and SOD-4 the extracellular space enzyme [20]. There are two NOXs of the Duox sub-class, with BLI-3 required for proper development of the cuticle via tyrosine cross-linking [21–23], and the very similar DUOX-2 to which no function has yet be assigned [24, 25].

One of the ways ROS can participate in signalling, is by modulating the activities of small GTPases. For examples, the Ras signalling pathway is known to be modulated by ROS in mammals [26, 27] and *C*. *elegans* [4, 28]. One of the ways the RAS pathway is affected by ROS in mammals is by direct oxidation of a sensitive cysteine (C118) of the RAS protein itself, by nitric oxide (NO) and superoxide [29, 30] as well as by hydrogen peroxide in the presence of transition metals [31]. This has been mostly studied in vitro [29–33], and in cultured cells [27, 34–36] but to a much lesser extent in vivo [37]. Redox-sensitive cysteines have also been studied in other small GTPases, such as Rac, where the C18 cysteine has been identified as redox-sensitive [38] as well as Rho, where it’s the C20 cysteine that is sensitive [39]. Studies with Ras and Rac have generally concluded that oxidation of these cysteines (C118 and C18, respectively) leads to the activation of the protein by stimulating guanine nucleotide release. In contrast, oxidation of C20 generally inhibits Rho proteins because of the presence of an additional cysteine, C16. Oxidation of C20 promotes guanine nucleotide release but subsequent disulfide bridge formation between C16 and C20 prevents guanine nucleotide binding, thus inactivating the protein [39]. A powerful tool to study the roles of these sensitive cysteines in vitro and in vivo is by replacement of the cysteine by a serine that cannot be oxidized [6, 33, 38] or by an aspartic acid to mimic oxidation [38, 40].

**Fig 1:**
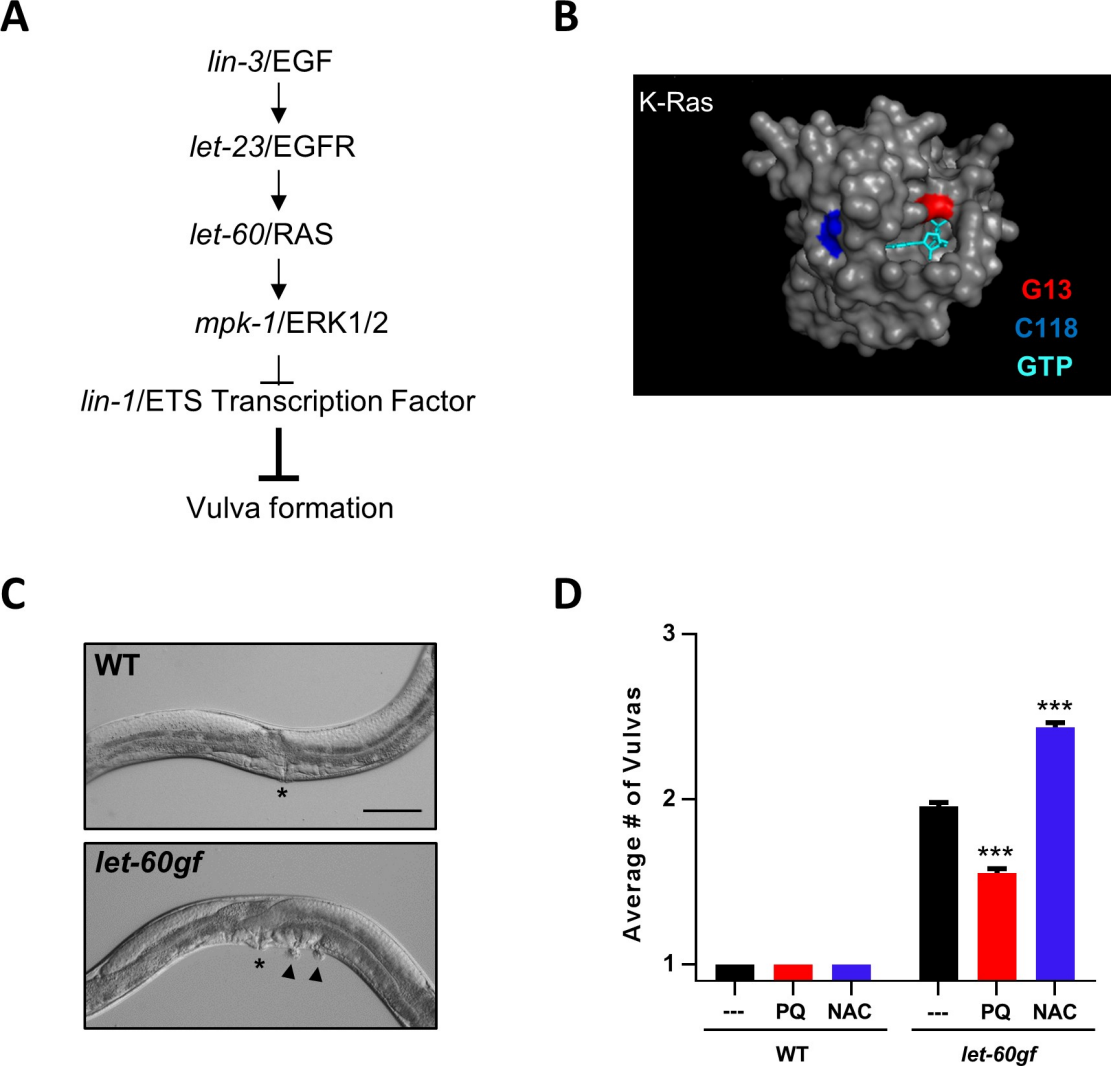
The multivulva phenotype of *let-60rasgf* is sensitive to ROS. *let-60ras(n1046gf)* is denoted in the figure as *let-60gf*. **A** The genetic pathway by which LET-60ras promotes vulval development. *C*. *elegans* gene names are in lowercase italics, and the corresponding mammalian protein homologues are in uppercase. The pathway depicts the genetic epistatic relationships between the genes rather than the biochemical interactions of the proteins. Gain-of-function mutations that activate LET-60ras or loss-of-function mutations in *lin-1* lead to the development of multiple vulvas. **B** Space-filling model of human K-Ras bound to GTP, showing the residue (G13) that is mutated in *let-60rasgf*, and the redox-sensitive cysteine (C118). The structure (PDB ID: 3GFT) was derived using Pymol. **C** Sample images from mutants scored in **D** (asterisks denote the vulvas and arrowheads the pseudovulvas). The scale bar represents 50 μm. **D** Number of vulvas of the wild type and the *let-60rasgf* mutant after treatment with PQ and NAC. ***P = 0.0001 compared to *let-60rasgf*.

In *C*. *elegans* the Ras pathway has been particularly well characterized for its role in the development of the *C*. *elegans* vulva, the egg-laying organ [41, 42](**Fig 1A**). *let-60ras* encodes the *C*. *elegan*s orthologue of mammalian Ras and is most similar to K-Ras [43]. Severe loss-of-function mutants of *let-60ras* cannot survive, but a gain-of-function mutation (*n1046gf*), resulting in a G13E substitution, is viable [44] (below we denote this mutation as *let-60rasgf*). Such oncogenic mutations at G13 (**Fig 1B and S1 Fig**) favor the active GTP-bound state [45, 46]. In *C*. *elegans*, this mutation induces the formation of multiple vulvas instead of only a single one: the multiple vulva phenotype (Muv) (**Fig 1C**). An important relay downstream of LET-60ras is MPK-1, the sole *C*. *elegans* homologue of the extracellular-signal regulated kinase (ERK1/2), whose level of phosphorylation at two sites is one of the molecular readouts of LET-60ras activation [47](**Fig 1A**). LIN-1, an ETS-domain transcription factor, acts downstream of MPK-1 to inhibit vulval cell fates [47, 48]. Whereas it is gain-of-function mutations in *let-60ras* that produce the Muv phenotype, it is loss-of-function mutations that do so in *lin-1*.

Here we use *C*. *elegans* vulva formation in the sensitized *let-60rasgf* gain-of-function background to dissect aspects of ROS signalling in a fully in vivo situation. We do this with genetic and pharmacological manipulations that are in the physiological range, with no impact on the organism’s health. We monitor real developmental outcomes (vulva formation) and, by controlling sources, sinks, and especially targets, we could identify genetically distinct ROS signalling pathways, despite that fact that they act in parallel and use the same enzymes and the same active molecules (superoxide and peroxide). We also provide strong evidence that it is hydrogen peroxide, rather than any other ROS species, that is responsible for cysteine oxidation and that in *C*. *elegans*, in vivo, oxidation inhibits, rather than activates LET-60ras and CED-10rac.

## Results

### RAS signalling is sensitive to ROS

The *let-60rasgf* mutant provides a sensitized background to score changes in RAS signalling, and allowed for extensive characterization of the pathway by identifying suppressors and enhancers [42]. We used the same logic to identify and characterize mechanisms of ROS signalling acting on the RAS pathway and on vulva formation in general. We treated *let-60rasgf* mutants with the prooxidant paraquat (PQ). PQ can potentially generate superoxide at many cellular sites [49], but its main site of superoxide production is believed to be in the mitochondrial matrix [50, 51]. We used a very low concentration of PQ (0.1–0.2 mM) that has been shown to increased mitochondrial superoxide [6, 52, 53] but without toxicity [52]. Such a low concentration might provide for alteration of ROS signals within the physiological range. In the presence of mitochondrial and cytoplasmic SODs, increased superoxide generation by PQ is expected to lead to increased hydrogen peroxide generation. N-acetyl cysteine (NAC) is a precursor of glutathione and thus functions as an antioxidant by facilitating the removal of peroxides, including hydrogen peroxide [54]. We used NAC at 9 mM, a concentration that has been shown to lower ROS [55] but has no effect on wild type viability [52]. Like other studies using prooxidants on *let-60rasgf* mutants [28], we found that although PQ had no effect on vulva formation in the wild type, it partially suppressed the Muv phenotype of *let-60rasgf* (**Fig 1D**). Conversely, we found that NAC treatment had no effect on the wild type but enhanced the Muv phenotype of *let-60rasgf* (**Fig 1D**). We tested two additional alleles of *let-60* in addition to the canonical *n1046gf* allele (**S2 Fig**). The *n1700* allele leads to the same amino acid change as *n1046* (G13E), but was independently isolated and is thus in a different background. It leads to a slightly more severe Muv phenotype. The effects of PQ and NAC on this allele were qualitatively the same, suppression and enhancement, respectively. However, the suppression was relatively greater and the enhancement relatively less, likely due to the more severe baseline Muv phenotype. We also tested *ga89*, a weaker temperature-sensitive allele [56]. However, the allele is so weak, even at the restrictive temperature (26°C), that only the suppressing effect of PQ could be reasonably inferred (**S2 Fig**). At this stage we could infer that oxidation inhibits a target that could be either LET-60ras itself or another activator of the RAS pathway. All numerical values for all Muv data shown in bar graphs in all figures are given in **S1 Table**. We report the total number of vulvas (which includes both the main vulva and the ectopic pseudovulvas) and the controls shown are always scored in parallel for every experiment.

### Cysteine C118 of LET-60ras is the target of oxidation by PQ

**Fig 2:**
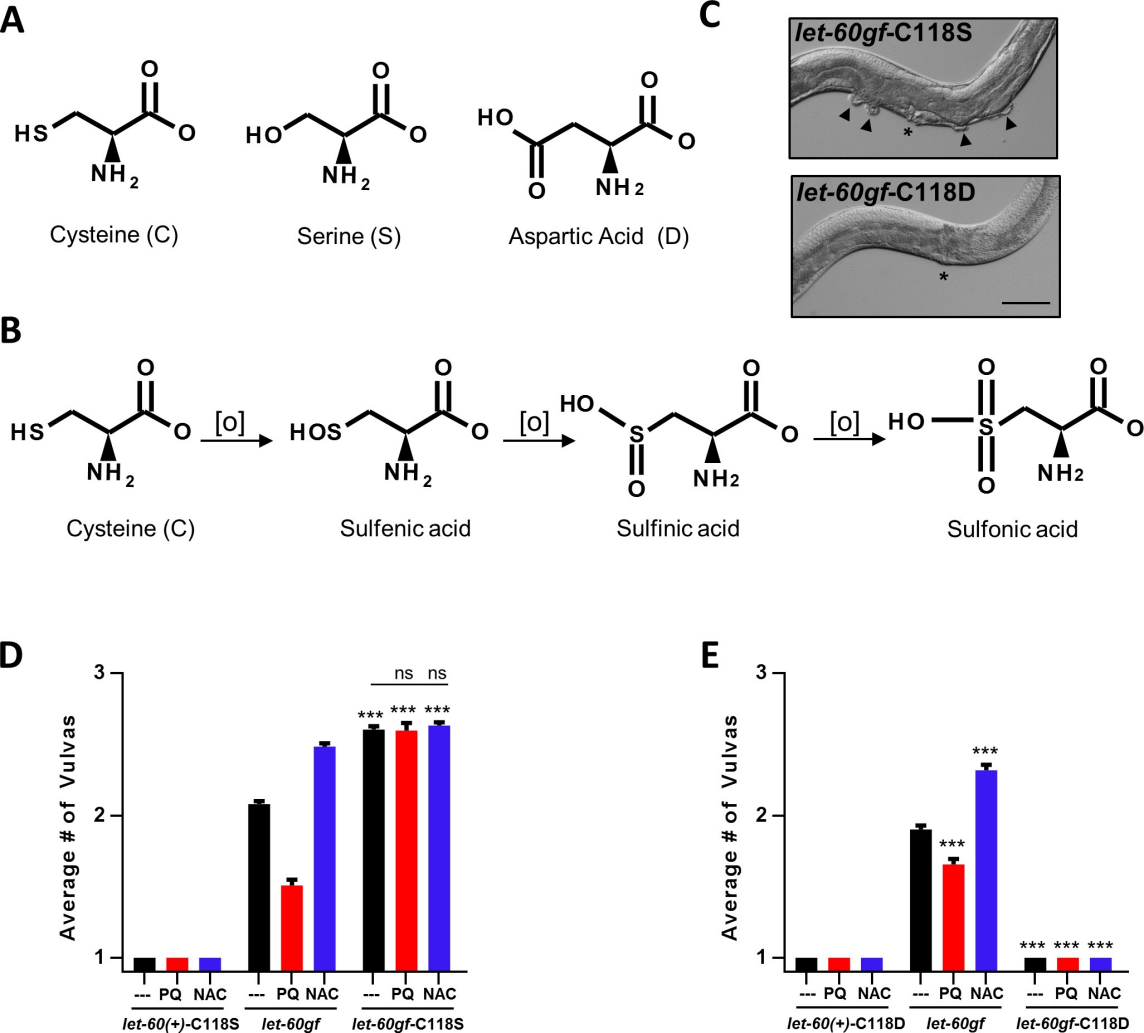
The activity of LET-60ras is inhibited by oxidation of C118. *let-60ras(n1046gf)* is denoted in the figure as *let-60gf*. **A** Serine mimics a non-oxidizable Cysteine, whereas Aspartic acid mimics Sulfinic acid, a doubly oxidised form of Cysteine. **B** Oxidative modifications of Cysteine. **C** Sample images from mutants scored in **D** and **E** (asterisks denote the vulvas and arrowheads the pseudovulvas). Scale bar represents 50 μm. **D, E** ***P = 0.0001 compared to *let-60rasgf* or as indicated. **D** C118S denotes a CRISPR modification which changed a TGT codon to a TCT thus resulting in a Cysteine to Serine substitution at amino acid position 118 (C118S). Number of vulvas of *let-60ras(+)*-C118S, *let-60rasgf*, and *let-60rasgf*-C118S mutants in combination with PQ and NAC treatments. **E** C118D denotes a CRISPR modification which changed a TGT codon to a GAT thus resulting in a Cysteine to Aspartic Acid substitution at amino acid position 118 (C118D). Number of vulvas of *let-60ras(+)*-C118D, *let-60rasgf*, and *let-60rasgf*-C118D mutants in combination with PQ and NAC treatments.

As described in the introduction, cysteine C118 of mammalian RAS is a known potential target of oxidation by NO and superoxide, with oxidation resulting in activation of the protein. Nitrosylation is not relevant in *C*. *elegans*, which lacks nitric oxide synthases [57]. We tested whether C118 was involved in the PQ and NAC sensitivity that we observed (although we observed inhibition rather than activation by PQ, and activation by an antioxidant, NAC). We used CRISPR to replace C118 with a serine (C118S). Serine has a similar structure to cysteine but cannot be oxidized by cellular ROS [58](**Fig 2A and 2B)**. We produced C118S alleles, *qm226* and *qm225*, in the wild type and *let-60rasgf* backgrounds, respectively. Below we also describe alleles in which C118 is replaced by aspartic acid (C118D). For clarity, we use the following formalism when new alleles that affect C118 are involved: we denotate the wild type *let-60* allele as *let-60(+)* and we use *let-60ras(+)*-C118S (or C118D) and *let-60rasgf*-C118S (or C118D) to denotate single and double mutants in which C118 has been replaced by another amino acid.

The C118S replacement had no effect on vulva formation in the wild type, but strongly enhanced vulval induction in *let-60rasgf* mutants (**Fig 2C and S3 Fig**). We scored both the Muv phenotype by counting visible pseudovulvas in adults (**Fig 2C and 2D**) as well as invaginations in L4-stage larvae (**S3 Fig**). The C118S replacement fully suppressed all effects by PQ or NAC (**Fig 2D)**. The degree of enhancement produced by C118S was very similar to that produced by NAC (**Fig 2D**). We conclude that in the *let-60rasgf* background, PQ acts on the Muv phenotype by increasing hydrogen peroxide levels, leading to increased oxidation of C118, and NAC acts by preventing oxidation of C118 by lowering hydrogen peroxide levels. When C118 is replaced by a serine that cannot be oxidized, neither compound has any effect. The fact that the C118S replacement leads to increased vulva formation indicates that C118 is normally partially oxidized. The picture of RAS oxidation by ROS is thus very different in living intact *C*. *elegans* from that in vertebrate cells: both the mechanism of oxidation (by hydrogen peroxide rather than by superoxide or nitric oxide) and the consequence of oxidation (inhibition rather than activation) are different.

### Mimicking constitutive oxidation of C118 fully suppresses the oncogenic *let-60rasgf* allele

Oxidation of cysteine produces cysteine sulfenic acid, which can be further oxidised to form sulfinic acid and then sulfonic acid [59](**Fig 2B**). The molecular shape and charges of cysteine sulfinic acid is mimicked by aspartic acid (D) [40](**Fig 2A and 2B**). We used CRISPR to replace C118 with aspartic acid (C118D), creating two alleles, *qm227* and *qm228*, in the *let-60rasgf* and in the wild-type backgrounds, respectively. This should mimic an intermediate but permanent degree of oxidization of C118 (**Fig 2B**). Strikingly, both *let-60ras(+)-*C118D and *let-60rasgf*-C118D mutants have only a single vulva and, like the wild type, are completely insensitive to PQ and NAC (**Fig 2C and 2E)**. This is consistent with our interpretation of the effects of PQ and NAC treatment on *let-60rasgf* and *let-60rasgf-*C118S: oxidation at C118 inhibits Ras signalling. Note that the down-regulation produced by the C118D substitution doesn’t prevent the formation of a vulva, whereas loss-of-function mutations in *let-60ras* are vulvaless [44]. Thus, one possibility for the complete suppression of the multi-vulva phenotype of the *let-60rasgf* oncogenic gain-of-function mutation (G13E) by C118D is that the mode of action of oncogenic mutations at G13 might be by interference with the normal regulation by oxidation at C118 (**Fig 1B**). In other words, the C118D mutation could be specifically counteracting the effects of the gain-of-function mutation rather than simply down-regulating LET-60rasgf activity. However, our findings of the level of expression of the LET-60rasgf-C118D protein suggest that there may be other possible reasons for the complete suppression of the Muv phenotype in these double mutations (see below).

### The degree of oxidation of C118 or the inhibition of oxidation by the C118S mutation does not act on vulva formation through MPK-1 phosphorylation

**Fig 3:**
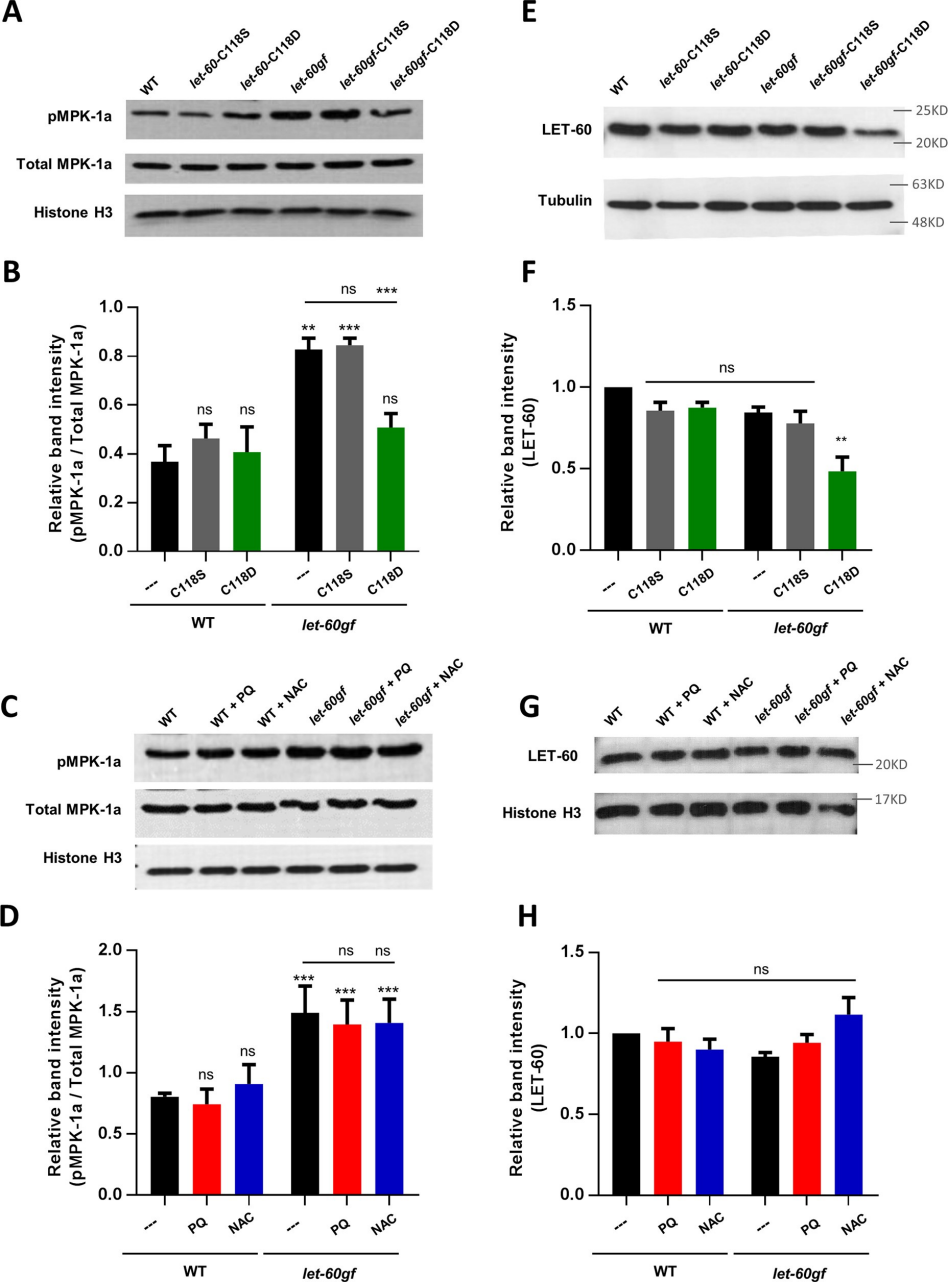
The effects of altered oxidation by PQ and NAC and the C118S mutation are not mediated via MPK-1 or by altered protein stability whereas the *let-60rasgf*-C118D double mutation affects both. **A** A representative Western blot for phosphorylated MPK-1a (pMPK-1a), total MPK-1a and Histone H3 as a loading control. Unprocessed original scans of blots and additional analyses are shown in S4 Fig. **B** The mean ratio of phosphorylated MPK-1a (pMPK-1a) to total MPK-1a ± SEM of three different Western blots. **C** A representative Western blot for phosphorylated MPK-1a (pMPK-1a), total MPK-1a and Histone H3 as a loading control. Unprocessed original scans of blots and additional analyses are shown in S5 Fig. **D** The mean ratio of phosphorylated MPK-1a (pMPK-1a) to total MPK-1a ± SEM of three different Western blots. **E** A representative Western blot for LET-60 with Tubulin as a loading control. Unprocessed original scans of blots are shown in S6A Fig. **F** The mean band intensity ± SEM of three different Western blots normalised to band intensities in the wild type. **G** A representative Western blot for LET-60 with Histone H3 as a loading control. Unprocessed original scans of blots are shown in S6B Fig. **H** The mean band intensity ± SEM of three different Western blots normalised to band intensities in the wild type. Molecular weight markers are shown for E and G as different loading controls were used for these blots. All bars are compared to the wild-type control bar or as indicated. **P<0.01, ***P<0.001.

Activation of the Ras pathway leads to increased phosphorylation of the downstream effector MPK-1 in *C*. *elegans* as in other systems [47, 60] (**Fig 1A**). To investigate whether changes of oxidation of LET-60rasgf by compound treatment or by mutations (C118S or C118D) counteracts or enhance the effects of *let-60rasgf* on MPK-1 phosphorylation, we used commercial antibodies to quantify MPK-1 phosphorylation in vivo, by Western Blot (**Fig 3A and 3B and S4 Fig**). There are two isoforms of MPK-1 (a and b), with MPK-1a believed to be the relevant isoform to signalling events in the animal’s soma, including vulval development [61]. Using a variety of controls to ensure accurate quantification (**S4 Fig and S5 Fig**), we observed increased phosphorylation of MPK-1a in *let-60rasgf* mutants (**Fig 3A and 3B**) as expected. However, phosphorylation of MPK-1a was not affected in any way in *let-60ras(+)*-C118S or *let-60rasgf-*C118S mutants and neither was it significantly suppressed by PQ treatment or enhanced by NAC treatment of *let-60rasgf*, despite the dramatic effects of the treatments and the mutation on the Muv phenotype (**Fig 3A–3D**). These findings suggest that activated Ras might impinge on vulva formation through more than one effector and ROS levels regulate vulval development independently of MPK-1 phosphorylation (see Discussion). However, in contrast to the lack of effects of the treatments and of the C118S mutation, the C118D mutation was capable of suppressing the increases level of MPK-1 phosphorylation that is observed in *let-60rasgf* mutants, such that the level of MPK-1 phosphorylation in *let-60rasgf-*C118D mutants is the same as that observed in the wild-type. See below for additional discussion of this observation.

### The effects of altered oxidation and the C118S mutation are not mediated by altered protein stability

We sought to investigate whether changes of protein stability could be the mechanism by which oxidation affects LET-60rasgf and RAS signaling. The possibility existed because overexpression of wild type LET-60ras can lead to the Muv phenotype [62]. We examined how the C118S mutation, and PQ and NAC treatments affect the LET-60ras protein levels for both LET-60ras(+) and LET-60rasgf using Ras-specific commercial antibodies (**Fig 3E–3H and S6 Fig).** We did not observe any effects on protein levels nor did we observe any effects on *let-60* mRNA levels (**S7 Fig**).

### The LET-60rasgf-C118D double mutation lowers protein levels

In contrast to what we observed with drug treatments and for LET-60rasgf-C118S, the protein level of LET-60rasgf-C118D but not of LET-60ras(+)-C118D is significantly lower than that of LET-60ras(+) (**Fig 3E and 3F**). Possibly therefore, the full suppression of the Muv phenotype observed in *let-60rasgf* -C118D mutants could be due to the lower level of protein acting on the MPK-1 phosphorylation pathway. Alternatively, the depth of the effect (complete suppression of Muv) could be the result of a double effect: an effect on the MPK-1 pathway via lower LET-60rasgf protein expression and an effect on the MPK-1-independent pathway that appears to mediate the other effects of changes to the oxidation status of C118 of LET-60ras.

### C118 oxidation depends on the cytoplasmic pool of hydrogen peroxide regulated by SOD-1

**Fig 4:**
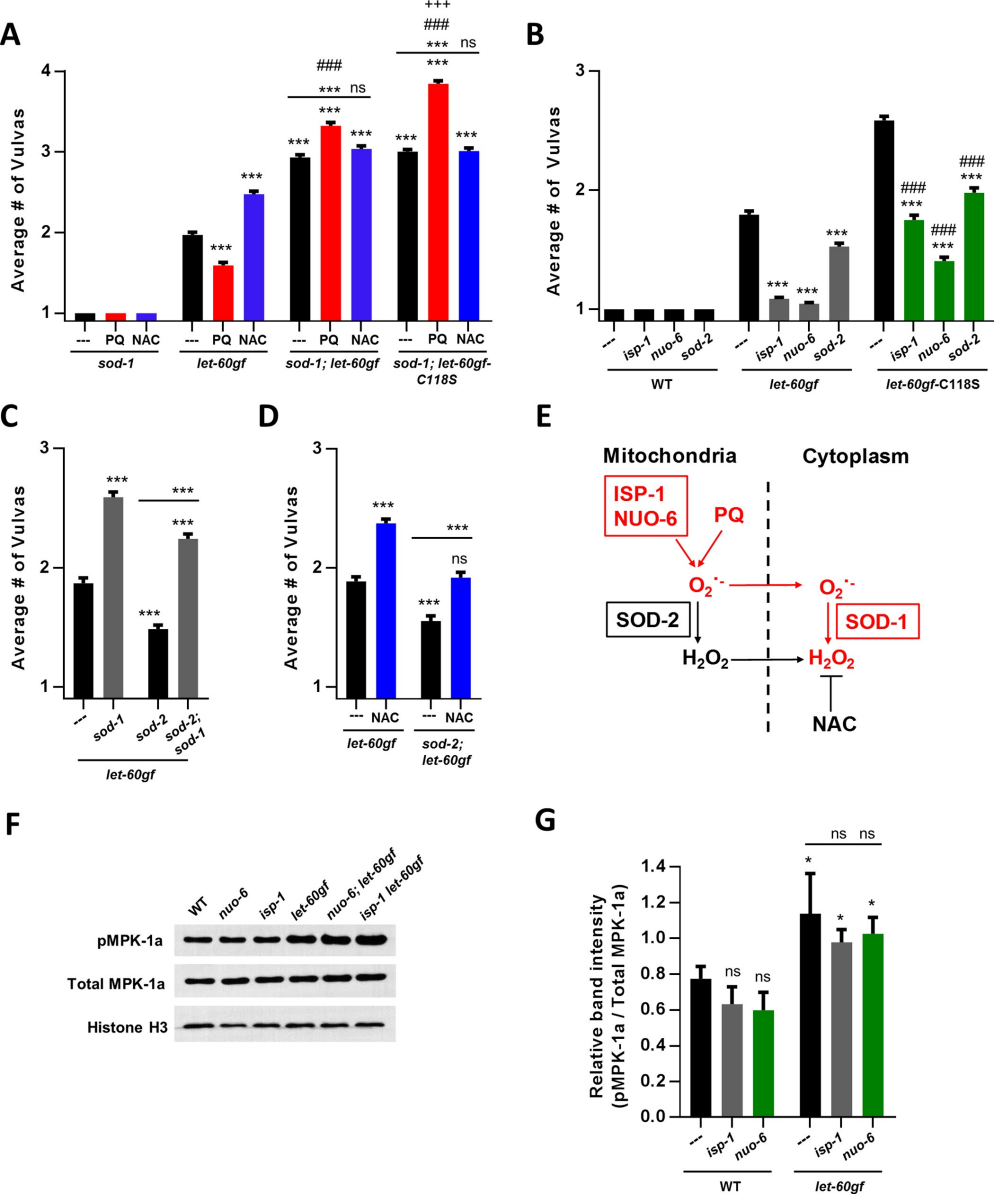
The SOD-1-dependent pool of cytoplasmic hydrogen peroxide regulates Ras via oxidation of C118. *let-60ras(n1046)gf* is denoted in the figure as *let-60gf*. ***P = 0.0001 compared to *let-60rasgf* or as indicated. **A** Number of vulvas of *sod-1*, *let-60rasgf*, *sod-1; let-60rasgf* and *sod-1; let-60rasgf-*C118S mutants in combination with PQ or NAC treatments. ###P = 0.0001 for PQ treatment compared to PQ treatment of *let-60rasgf*. +++P = 0.0001 for PQ treatment of *sod-1; let-60rasgf-*C118S compared to PQ treatment of *sod-1; let-60rasgf*. **B** Number of vulvas of long-lived mitochondrial mutants in an otherwise wild-type background and in combination with the *let-60rasgf* mutant, with and without the C118S mutation. ***P = 0.0001 compared to *let-60rasgf* for the first set of bars, and compared to *let-60rasgf-*C118S for the second set of bars. ###P = 0.0001 compared to the mutation in the *let-60rasgf* background. **C** Number of vulvas of *sod-2* mutants in combination with *sod-1* and the *let-60rasgf* mutation. **D** Number of vulvas of *sod-2* mutants, in combination with the *let-60rasgf* mutation and NAC treatment. **E** A model that suggests a path by which PQ, SOD-1, and the loss of SOD-2, all increase cytoplasmic H_2_O_2_ and thereby suppress *let-60rasgf*. For simplicity SOD-1 is shown in the cytoplasm although it may also be in the mitochondrial inter-membrane space. **F** A representative Western blot for phosphorylated MPK-1a (pMPK-1a), total MPK-1a and Histone H3 as a loading control. Unprocessed original scans of blots and additional analyses are shown in S8 Fig. **G** The mean ratio of phosphorylated MPK-1a (pMPK-1a) to total MPK-1a ± SEM of three different Western blots. All bars are compared to the wild-type control bar. *P<0.01.

Any excess O_2_^•-^ produced by PQ treatment is expected to be converted to hydrogen peroxide by superoxide dismutases. Although there are 5 SODs in *C*. *elegans*, cytoplasmic SOD-1 and mitochondrial SOD-2 account for virtually all SOD activity [20, 63]. SOD-1 may also be present in the mitochondrial inter-membrane space as is the case in other organisms [64]. To determine the enzymatic source of the relevant hydrogen peroxide, we constructed a *let-60rasgf;sod-1* double mutant. These double mutant animals displayed an enhanced Muv phenotype (**Fig 4A**), similar to that obtained by treating *let-60rasgf* with NAC or replacing cysteine C118 with serine (**Fig 2D**). Furthermore, the mutants’ Muv phenotype is no longer enhanced by NAC or suppressed by PQ (**Fig 4A**). These observations suggest two, not mutually exclusive, mechanisms concerning the origin of the hydrogen peroxide that can oxidize C118: 1) Wherever it is produced, the superoxide produced by PQ reaches the compartment in which SOD-1 is present, and/or 2) in the absence of SOD-1, any hydrogen peroxide produce by other SODs from PQ-dependent superoxide cannot produce a sufficient elevation of cytoplasmic hydrogen peroxide to affect C118 of LET-60ras. Other elements of the data presented in **Fig 4A**, such as the enhanced vulva formation produced by PQ and the effect of the C118S mutation in the absence of SOD-1, are discussed further below.

### Ras signalling is sensitive to increased mitochondrial superoxide levels in mitochondrial mutants

Mitochondria are a site of ROS formation that has been studied extensively. Mitochondrially-derived ROS are believed to be involved in signals that affect mitochondrial dynamics [65], mitophagy and autophagy [66], apoptosis, responses to changes in oxygen levels particularly hypoxia, inflammatory responses [67, 68], wound healing [6, 69] and aging [70]. We used three mutations that have elevated mitochondrial superoxide levels. *sod-2* mutants completely lack the main mitochondrial matrix superoxide dismutase SOD-2 [71]. *isp-1* and *nuo-6* are point mutants in subunits of the mitochondrial respiratory chain that lead to low electron transport, low ATP levels but high level of superoxide generation [72, 73]. All three mutations suppress the Muv phenotypes in double mutant combinations with *let-60rasgf* (**Fig 4B**). *sod-2* suppresses by about 40% but *isp-1* and *nuo-6* suppresses almost completely (**Fig 4B**).

As described above, the loss of SOD-1 enhances the Muv phenotype (**Fig 4A**) while the loss of SOD-2 suppresses it (**Fig 4B**). We found that suppression of the Muv phenotype by loss of SOD-2 is partially abrogated by the loss of SOD-1 (**Fig 4C**) or by treatment with NAC (**Fig 4D**). These observations suggest that loss of mitochondrial SOD-2 suppresses *let-60rasgf* via a SOD-1-dependent increase in cytoplasmic or mitochondrial inter-membrane hydrogen peroxide (**Fig 4E)**. This conclusion was supported by the finding that replacement of C118 with serine in the *let-60rasgf* background suppresses the effect of loss of SOD-2 on the Muv phenotype (**Fig 4B**). **Fig 4E** suggests a model of how the increased superoxide produced in the mitochondrial matrix in the absence of SOD-2 reaches the inter-membrane space and/or cytoplasm, where SOD-1 is located. The model includes that in the absence of a mitochondrial matrix SOD, superoxide can be transported out of both mitochondrial compartments into the cytoplasm through specialised channels [74, 75]. Thus, our results suggest that in the absence of SOD-2, superoxide exits the mitochondria to raise levels of hydrogen peroxide in the inter-membrane space and/or the cytoplasm through the action of SOD-1.

Similarly, we tested whether the Muv suppression by *isp-1* and *nuo-6* depended on C118 by scoring the Muv phenotype in *isp-1 let-60rasgf*-C118S and *nuo-6; let-60rasgf*-C118S double mutants (**Fig 4B**). The C118S replacement also suppressed the Muv suppression by these mitochondrial mutants. Since functional wild-type SOD-2 is present in *isp-1* and *nuo-6* mutants the effect of their known increased superoxide generation might act through a SOD-2-dependent production of hydrogen peroxide in the mitochondria. This excess mitochondrial hydrogen peroxide could participate to the cytoplasmic pool by exiting the mitochondria through passive membrane diffusion or through specialized channels [76] (**Fig 4E**). Consistent with what we observed for *let-60ras(+)-*C118S and *let-60rasgf-*C118S (**Fig 3C and 3D**), the *isp-1* and *nuo-6* mutations do not affect the levels of MPK-1 phosphorylation (**Fig 4F and 4G and S8 Fig**). In addition, the fact that the suppression of the effect of the ETC mutants by C118S is not complete suggests that other consequences of the mutations, such as low electron transport and low ATP levels might also participate in suppressing the Muv phenotype.

For our observations on the effect of *sod-2*, *isp-1*, and *nuo-6* on the Muv phenotype we cannot exclude an alternative interpretation: that all the effects we observe are additive, rather than revealing interactions. However, given the known relationships between the cytoplasmic and mitochondrial ROS pools discussed and reviewed in the above paragraphs, this alternative model appears less likely.

### The intracellular superoxide pool affects vulva formation by a separate pathway that is independent from hydrogen peroxide generation

As we have seen above, the C118S mutation produces an elevated Muv phenotype that is very similar to that produced by NAC, and NAC has no additional effects on C118S mutants. In addition, the suppression of the Muv phenotype by PQ is fully abolished by the C118S mutation (**Fig 2D**). Thus, *let-60rasgf-*C118S mutants, with or without NAC or PQ treatment, have almost the same Muv phenotype as *let-60rasgf* treated with NAC (**Fig 2D**). However, loss of SOD-1 in the *let-60rasgf* background produces an increase in the Muv phenotype that is significantly greater than that produced by NAC (**Fig 4A**). Furthermore, in the absence of SOD-1, PQ not only fails to suppress the Muv phenotype, it actually increases it (**Fig 4A**). In addition, the Muv increasing effect of PQ in the *sod-1* background is even stronger in the absence of C118 in *sod-1;let-60rasgf-*C118S mutants (**Fig 4A**). Thus, increased superoxide formation by PQ in the absence of SOD-1 (which would convert it to hydrogen peroxide), and in the absence of a target for hydrogen peroxide (in the *let-60rasgf*-C118S background), leads to a stimulation of the Muv phenotype. Note that the fact that PQ simulates more in the *let-60rasgf-*C118S background means that there is likely residual hydrogen peroxide formation from PQ-dependent superoxide even in the absence of SOD-1. This residual hydrogen peroxide can act in an inhibitory fashion on C118. This effect is eliminated in the C118S mutant leading to even greater vulva formation under the action of PQ. Together, the observations presented in **Fig 4A** indicate the existence of a distinct pathway in which superoxide stimulates the Muv phenotype, independently of both SOD-1-dependent hydrogen peroxide formation and oxidation of C118.

### The superoxide-stimulated pathway affects vulval development by acting downstream of LIN-1

**Fig 5:**
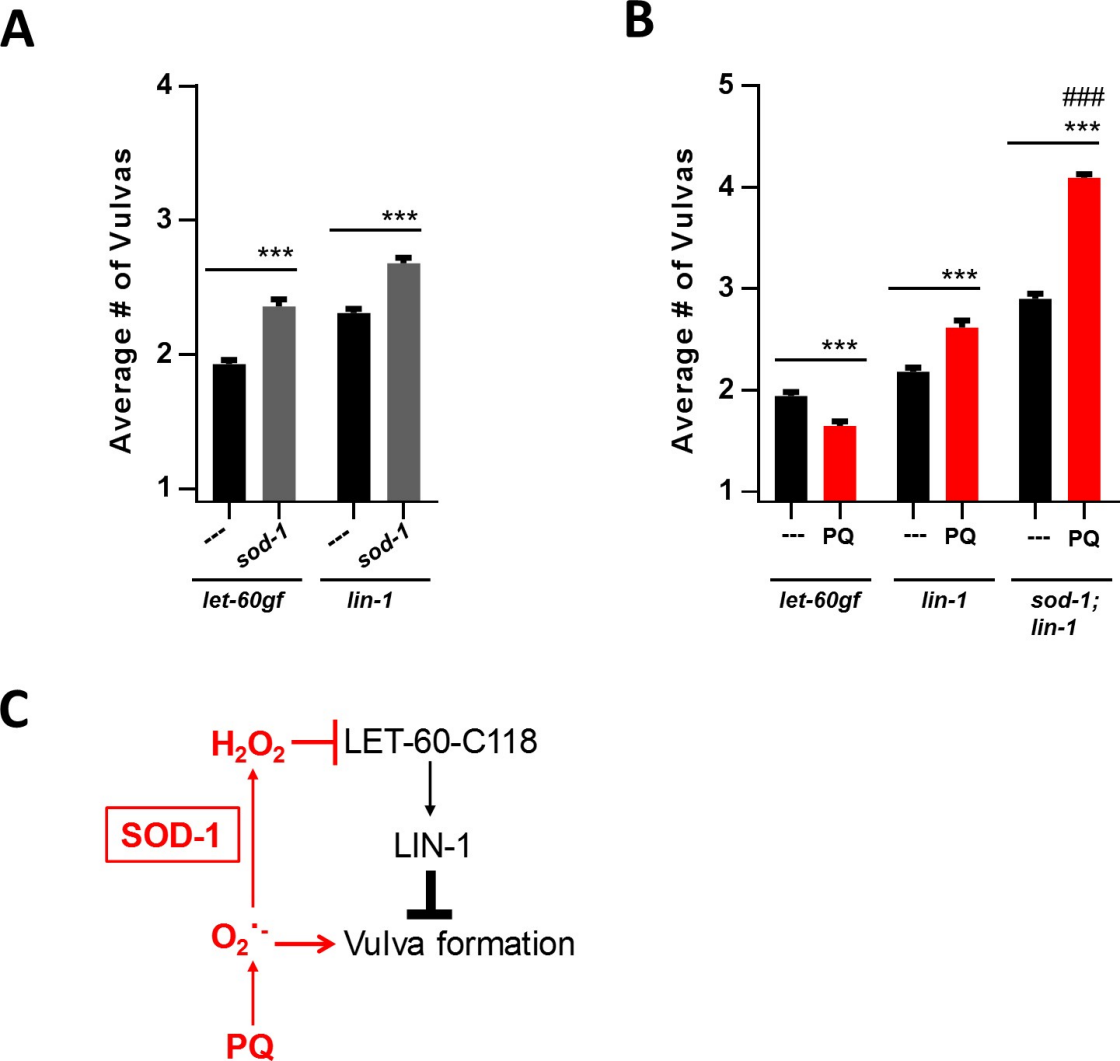
The intracellular pool of superoxide affects vulva formation by a pathway that acts downstream of *lin-1*. *let-60rasgf* is denoted in the figure as *let-60gf*. ***P = 0.0001 compared to control as indicated. **A** Number of vulvas of *sod-1* mutants in combination with the *let-60rasgf* and *lin-1* mutations. **B** Number of vulvas of *sod-1* mutants in combination with the *let-60rasgf* and *lin-1* mutations and PQ. ### P = 0.0001 for the effect of PQ treatment compared to *lin-1* with PQ treatment. **C** A model depicting the pathways by which cytoplasmic ROS affects vulval formation. Hydrogen peroxide inhibits vulval development by inhibiting LET-60 via oxidation of C118; superoxide promotes vulval development by acting downstream of LIN-1, on an unidentified target.

*let-60ras*-dependent signalling is implemented by the ETS-transcription factor LIN-1 [48] (**Fig 1A**). For vulval development loss of LIN-1 is epistatic to changes in the LET-60ras-dependent pathway. Thus, processes that act downstream of LIN-1 to affect the Muv phenotype are likely to be at least partially epistatic to LET-60ras signalling. We observed that loss of SOD-1 has the same effect on *lin-1* mutants as on *let-60rasgf* mutants, that is, it enhances the Muv phenotype (**Fig 5A**). However, PQ treatment has opposite effects on *let-60rasgf* and *lin-1* mutants: while it suppresses the Muv phenotype of *let-60rasgf* it enhances *lin-1* (**Fig 5B**). The effect of PQ is also additive to the effect of *sod-1* (**Fig 5B**), which is the same pattern we observed for *let-60rasgf* in **Fig 4A**. Thus, an increase in intracellular superoxide resulting from loss of SOD-1 appears to stimulate a second pathway, independent of C118 of *let-60ras* and acting downstream of *lin-1* (**Fig 5C**).

### The two *C*. *elegans* NADPH oxidases, BLI-3 and DUOX-2 affect vulva formation

**Fig 6:**
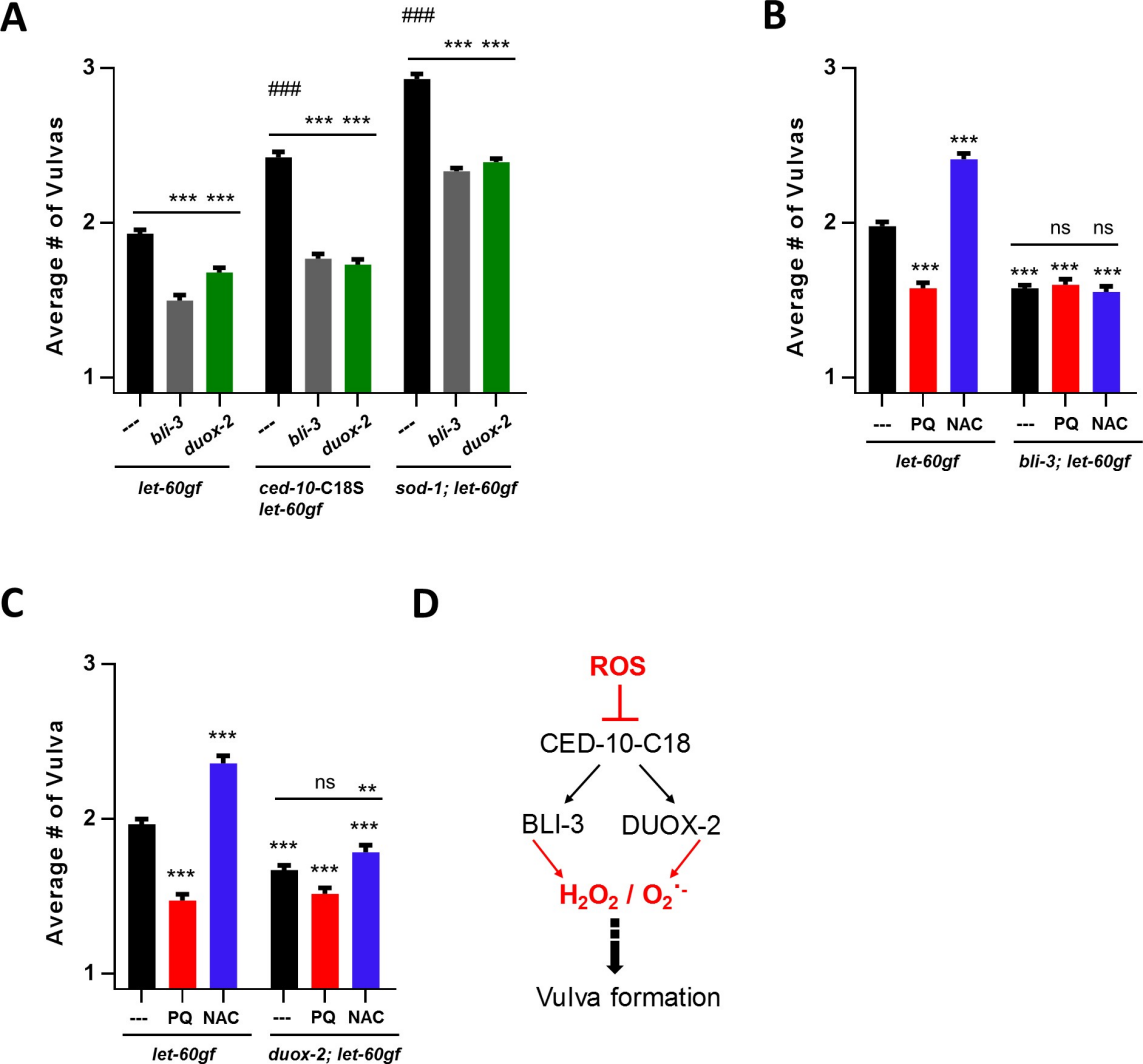
The NADPH oxidases BLI-3 and DUOX-2 affect vulva formation in a ROS-regulated manner. *let-60rasgf* is denoted in the figure as *let-60gf*. ****P = 0.0001 compared to *let-60rasgf* or control as indicated. **A** Number of vulvas of *bli-3* and *duox-2* mutants in combination with the *let-60rasgf* mutation alone, and in combination with *ced-10rac*-C18S and *sod-1* mutations. ###P = 0.0001 for *ced-10*-C18S *let-60gf* and *sod-1; let-60rasgf* compared to *let-60rasgf*. **B** Number of vulvas of *bli-3* mutants in combination with the *let-60rasgf* mutation, PQ and NAC. **C** Number of vulvas of *duox-2* mutants in combination with the *let-60rasgf* mutation, PQ and NAC. **D** Model illustrating how BLI-3 and DUOX-2 promote vulval formation. *ced-10rac* activates the NOXs, which promote vulva formation through the production of hydrogen peroxide, and perhaps also superoxide. These ROS targets have not been identified. *ced-10rac* is inhibited by ROS via C18. Neither the source nor the nature of this ROS is known.

NADPH oxidases (NOXs) are the main enzymatic sources of ROS in the cell. As described in the introduction, NOXs are membrane-bound enzymes that produce superoxide. The Duox sub-class possesses an additional peroxidase domain and can thus convert superoxide into hydrogen peroxide. While DUOXs have well-established roles at the plasma membrane, there is evidence that they may also have intracellular roles, such as at the ER-membrane (reviewed in [25]). The *C*. *elegans* genome encodes for two DUOX proteins: BLI-3 and DUOX-2. BLI-3 is expressed in the hypodermis, where it is required for cross-linking of collagen to form the cuticle. Complete loss of *bli-3* is lethal while reduction-of-function mutations in *bli-3* lead to a blistered cuticle phenotype (Bli), molting defects and altered pathogen susceptibility [25, 77]. Significant expression of *duox-2* has not been observed, and loss of *duox-2* has not previously been associated with any phenotype [25]. In order to test whether NOX-derived ROS participates in vulva formation, we examined how mutants of the *C*. *elegans* BLI-3 and DUOX-2 affect the *let-60rasgf* Muv phenotype. The *bli-3* mutation we used (*e767*) is in the peroxidase domain, and thus likely prevents hydrogen peroxide formation but not superoxide formation by BLI-3 [23]. The *duox-2(ok1775)* mutation is likely a strong loss-of-function mutation. We found that each of the NOX mutations leads to strong suppression of the Muv phenotype of *let-60rasgf* in double mutants (**Fig 6A**), suggesting that the wild-type function of both NOXs participate in stimulating the Muv phenotype. This is the first function that can be ascribed to DUOX-2. In addition to suppressing the Muv phenotype of *let-60rasgf*, loss of either of the NOXs renders the *let-60rasgf* mutants almost insensitive to NAC and PQ (**Fig 6B and 6C**). This indicates that the effect of the NOX mutations is epistatic to the effect of hydrogen peroxide on LET-60rasgf. However, we have not been able to link NOX activation to the elevation of the SOD-1-dependent intracellular superoxide pool that acts downstream on *lin-1* (**Fig 5**) (see below).

### Evidence for the activation of the NOX pathway by ROS

The activity of NOX enzymes is stimulated by the small GTPase Rac [78]. There are three Rac-like GTPases in *C*. *elegans* (CED-10, MIG-2, and RAC-2/3), although it is not certain that RAC-2/3 is a functional protein [79]. CED-10 and MIG-2 regulate cytoskeletal dynamics and function in multiple processes in the *C*. *elegans*, including phagocytosis of cell corpses, cell migration, axon pathfinding and growth cone protrusion [79]. *ced-10* and *rac-2* mutants have defects in vulval development, which can mainly be attributed to their roles in vulval cell migrations, but they also have a weak, synthetic defect in vulval cell specification [80]. Rac GTPases can be regulated by ROS. In particular, oxidation of cysteine C18 (**S9 Fig**) has been found to lead to activation of Rac1 in vitro and in cultured mammalian cells [38]. In light of this, we focused on the Rac1 homologue *ced-10* and used CRISPR to create a C18S allele, *ced-10rac(qm229)*, which should be insensitive to ROS. This allele significantly enhanced the Muv phenotype in the *let-60rasgf* background (**Fig 6A**). This was the reverse of the effect expected from mammalian studies, as we would expect that inhibition of CED-10 would phenocopy *duox-2* and *bli-3* loss-of-function mutations, but it had the opposite effect. We interpret this to mean that oxidation of C18 of CED-10 inhibits the protein. This would be similar to what we observed for C118 of LET-60. In both cases, we observed that the loss of the cysteine leads to activation rather than to inhibition of the activity. Since the C18S mutation had the opposite effect to that of the *duox-2* and *bli-3* mutations, we tested whether Rac was acting through each of the *C*. *elegans* Duoxs by creating *bli-3*(*e767*); *ced-10rac(qm229)* and *duox-2(ok1775); ced-10rac(qm229)* double mutants. The enhancement by C18S was abolished by either of the NOX mutations (**Fig 6A**), suggesting that the C18S replacement stimulates the Muv phenotype through both of the NOXs (**Fig 6D**).

**Fig 7:**
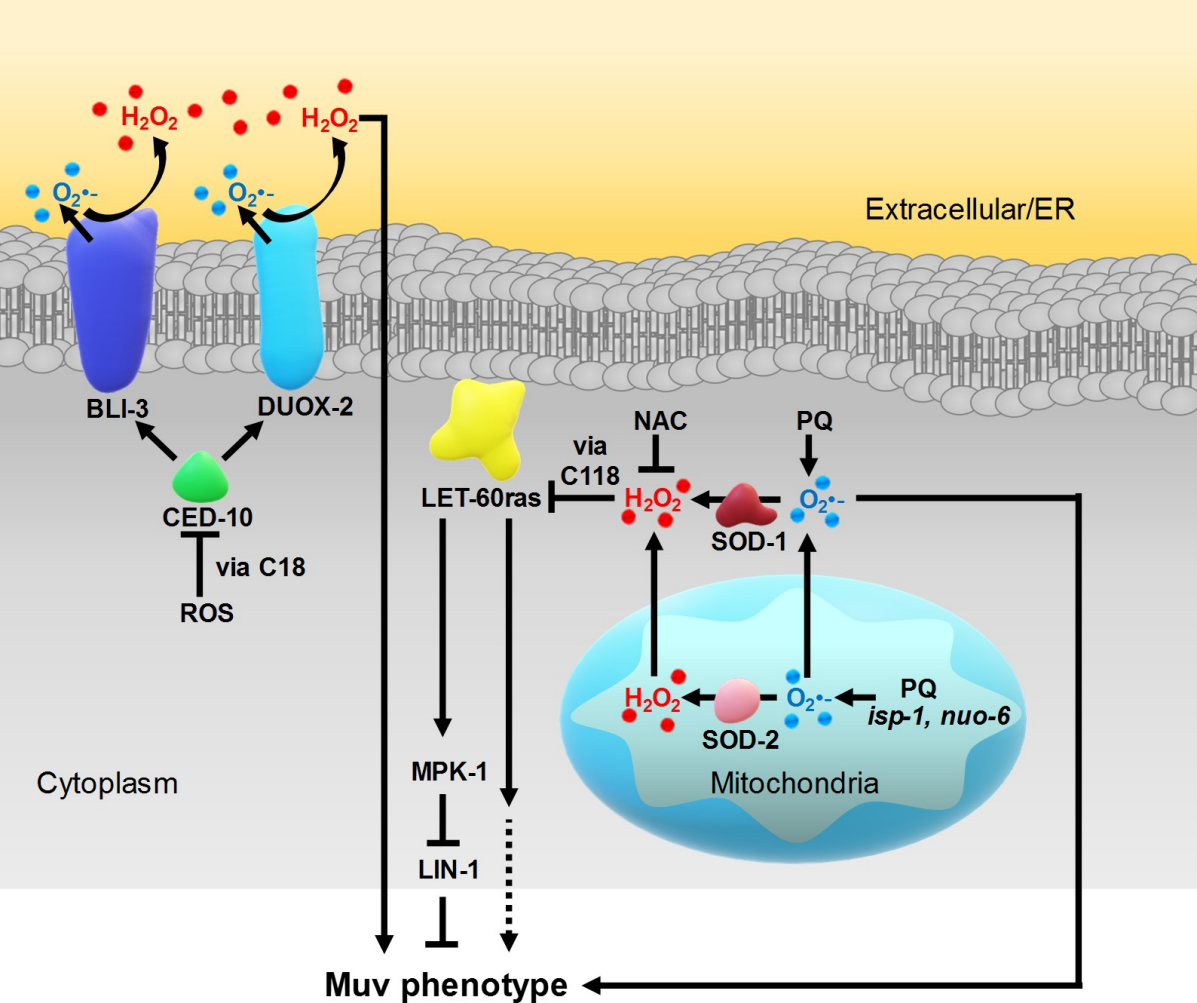
A model depicting three ROS-signalling pathways that affect vulval formation. Cytoplasmic hydrogen peroxide inhibits vulva development by acting directly on LET-60ras, via oxidation of C118. The state of oxidation of C118 is affecting vulva formation independently from MPK-1 phosphorylation via an as yet uncharacterized pathway. The source of hydrogen peroxide is SOD-1 and is either converted from cytoplasmic superoxide or superoxide that exits the mitochondria in the absence of the mitochondrial superoxide dismutase SOD-2. SOD-2-dependent hydrogen peroxide produced in the mitochondria can also participate in the SOD-1-dependent cytoplasmic pool. Cytoplasmic superoxide inhibits vulval development by acting downstream of LIN-1, via an unknown target. The NADPH oxidases BLI-3 and DUOX-2 promote vulva development by an independent pathway. This pathway is ROS-dependent as the NADPH oxidases are activated by CED-10Rac, which is inhibited by ROS via C18, however, the source and nature of this ROS are not known. The targets of NADPH oxidase-produced ROS have not been identified. It is also not known whether for vulva development NADPH oxidases act at the plasma membrane and produce ROS extracellularly, or whether they act at the ER membrane and produce ROS in the ER. All pathways are depicted as acting in the same cell although it is possible that the different signalling pathways act in different cell types and could thereby participate in intercellular communication during vulva development.

Next we wondered whether the increased superoxide due to loss of SOD-1, which stimulates the Muv phenotype (**Fig 4A**), acted through CED-10rac. To test for this we constructed the *let-60gf; sod-1;bli-3 and let-60gf;sod-1;duox-2* triple mutants. However, the effects of loss of SOD-1 and that of the NOX mutations were additive (**Fig 6A**). In any case, oxidation of C18 of CED-10rac would be inhibitory (as revealed by the activating effect of C18S), but the loss of SOD-1 is activating (**Fig 4A**). Thus, if there was a link between the effect of loss of SOD-1 and activation of the NOXs it would need to include additional relays, which would remain to be identified. Thus, we consider the NOXs to promote vulval formation in a ROS-regulated manner, by acting in a pathway that is parallel to both LET-60ras and to intracellular superoxide (**Fig 7**).

## Discussion

Our observations demonstrate that, by using molecular genetics, ROS signalling can be studied in a fully in vivo situation with the same detail as other forms of signal transduction. Findings in other systems such as yeast [81] and with other pathways such as the UPR^ER^ in *C*. *elegans* [5], reinforce this conclusion. ROS signalling resembles signalling through cAMP in that diffusible messages (ROS species) have sources and sinks. It resembles Ca^++^ signalling in its clear-cut compartmentalization, and it resembles phosphorylation in reversibly targeting specific residues and thus altering protein function. This last feature is particularly powerful for molecular genetic dissection of ROS signalling pathways and functions. There have been many efforts to obtain fluorescent probes for visualizing ROS in living cells similar to tools that allow to visualize calcium [82]. However, for many applications these techniques suffer from lack of resolution and specificity. Although better tools for visualization would be highly desirable, our findings demonstrate that detailed information about ROS signalling can be obtained despite the lack of sufficiently powerful visualization tools.

Our findings suggest that ROS produced in mitochondria can participate in cytoplasmic ROS signalling in multiple ways (**Figs 4E and 7**). In the presence of mitochondrial superoxide dismutase (SOD-2), superoxide produced in the mitochondrial matrix is converted to hydrogen peroxide and can contribute to the cytoplasmic pool of this species. In the absence of SOD-2, superoxide exits the mitochondrial matrix, where it is converted to hydrogen peroxide by the cytoplasmic superoxide dismutase (SOD-1) and participates in ROS signalling in the cytoplasm. At this stage we don’t know whether hydrogen peroxide directly modifies C118 as observed with other redox sensitive cysteines [83], or whether it is required for oxidation via additional steps.

In addition to a role for hydrogen peroxide, we also identified a specific signalling role for superoxide (**Figs 5C and 7**). By elevating the cytoplasmic superoxide pool by removal of SOD-1, with or without concomitant PQ treatment to enhance the effect, we showed that it can affect the Muv phenotype downstream of LIN-1, by acting on an as yet unidentified target. Interestingly, Xu and Chisholm have also demonstrated a specific role for superoxide in *C*. *elegans* wound healing. They showed that mitochondrial superoxide targets the *C*. *elegans* Rho GTPase RHO-1 [6]. Epidermal wounding causes a local increase in mitochondrial superoxide, which inhibits RHO-1 via the redox sensitive cysteines C16 and C20, and this promotes actin-dependent wound closure.

The NOX system is a transmembrane signalling system that can release ROS extracellularly or intracellularly [12] including in *C*. *elegans* [5, 25] We found that the activity of the NOXs could affect vulva formation independently of ROS regulation of LET-60rasgf via C118 (**Figs 6B, 6C and 7**). At this stage we don’t know if for this function the NOXs act intracellularly or extracellularly. The NOXs appear themselves to be regulated by intracellular ROS via oxidation of C18 of CED-10rac (**Fig 6A and 6D**). However, the species (e.g. superoxide or hydrogen peroxide) and the origin of the ROS that regulate CED-10rac, and thus the NOXs, is still unknown. We have also yet to identify the direct targets of cytoplasmic superoxide, and the targets of the NOX-derived ROS.

We identified two key targets of cytoplasmic ROS regulation of vulva formation as known redox sensitive cysteines in the small GTPases LET-60ras (C118) and CED-10rac (C18). Strikingly, we observed that in *C*. *elegans*, oxidation of C118 in LET-60ras and C18 in CED-10rac is inhibitory, while in vitro and cell culture experiments in mammalian cells have suggested they would be stimulatory by promoting guanine nucleotide dissociation. How to explain these differences? One important difference is that *C*. *elegans* does not produce, and likely doesn’t use, nitric oxide (NO) [57]. Thus, the cysteines that are susceptible to oxidation by NO are free to be oxidized by different reactive species, which might result in different effects on the target protein. It is also possible that the cellular redox conditions are different in *C*. *elegans* and mammalian cells, including differences in the concentration of various ROS species or the amplitude of variations in these concentrations. More generally redox conditions in vivo, in *C*. *elegans* and in mammals, might be very different from those in cultured cells. For example, most mammalian cell types experience low levels of oxygen, low levels of hormonal stimulation, and possibly very low or no amounts of NO. Thus, some of the findings in mammalian cells might be only pertain to the conditions in cultured cells. In contrast, our tools to manipulate ROS levels are all compatible with normal or extended survival of the animals.

In addition, the cellular redox conditions (as well as the composition of the cellular milieu in the direct vicinity of a ROS target) will contribute to determining whether a modification is inhibitory or stimulatory. That is to say, reactive nitrogen, superoxide and hydrogen peroxide promote Ras guanine nucleotide dissociation. But this by itself is not activating, it’s only activating under conditions that allow Ras to be competent to re-associate with GTP. Of note, in vitro, prolonged exposure of Ras in the presence of oxidants and GTP do not result in significant fraction of GTP-bound Ras, unless a radical scavenger is also included in the reaction [31].

We observed that PQ and NAC treatment and the C118S mutation, as well as *isp-1* and *nuo-6* mutations, have no effect on the enhanced MPK-1 phosphorylation resulting from the *n1046gf* allele, suggesting that the state of oxidation of LET-60ras affects a pathway that does not affect MPK-1 phosphorylation but that modulates vulva formation in the presence of elevated MPK-1 phosphorylation. We have no information yet as to the nature of this pathway. However, there is other evidence that such pathways may exist. For example, *let-60ras* mutants have many phenotypes in addition to vulval development, that indicate that LET-60ras acts in multiple cell types and tissues. *mpk-1* mutants share most of these phenotypes, suggesting that LET-60ras mainly signals through MPK-1 [47]. However, there are also some *let-60ras* phenotypes which are either not shared by *mpk-1* mutants, or for which an involvement of *mpk-1* has not been examined [42, 47], suggesting that there may be other effectors of LET-60ras. Even for vulval development, where it has been clearly established that LET-60ras acts via MPK-1 [84], it has also recently been shown that there is also MPK-1-independent signaling. In some VPCs LET-60ras acts through an alternative pathway, via RGL-1(RalGEF) and RAL-1(Ral), to specify the secondary vulval cell fate [85].

The possibility that mimicking constitutive oxidation of C118 suppresses a classical gain-of-function allele similar to oncogenic alleles in mammals is potentially of great interest. It suggests that the reason why mutations in the N-terminal G12 and G13 of Ras produce a gain-of-function could be intimately linked to events that involve the oxidation of C118. When C118 looks like it is permanently oxidized (with the C118D mutation) the G13E oncogenic mutation is fully suppressed. On the other hand, we found that the LET-60rasgf-C118D double mutant protein is expressed at a significantly lower level than LET-60rasgf. This lower level might be the basis for the complete suppression of the effect of *n1046gf* on MPK-1. However, given that PQ has no effect on protein levels or MPK-1 phosphorylation, we favor the model that both effects (the mimicking of constitutive strong oxidation and low level of expression) might be necessary for the complete suppression conferred by the C118D mutation. Identification of the hypothetical pathway downstream of LET-60ras oxidation could help resolve this issue in the future.

## Materials and methods

### General methods and strains

All animals were grown at 20°C and cultured on NGM plates. The Bristol strain N2 was used as the wild type. The mutations used in this study are as follows: *nuo-6(qm200)*, *sod-2(ok1030)*, *bli-3(e767)*, *duox-2(ok1775)* I, *sod-1(tm783)* II, *isp-1(qm150)*, *let-60(n1046)*, *let-60(n1700)*, *let-60(ga89)* and *lin-1(e1026)* IV. For temperature shift experiments, *let-60(ga89)* adults were switch to the restrictive temperature (26°C) as adults and the progeny was used for the assay.

For testing the effects of Methyl viologen dichloride hydrate/Paraquat (PQ; Sigma-Aldrich 856177) and N-Acetyl-Cysteine (NAC; Sigma-Aldrich A7250), compounds were dissolved in water and stored at 4°C. The compounds were added to the NGM just before pouring the plates. The final concentrations of PQ and NAC used for vulva studies were 0.1 mM and 9 mM, respectively.

### CRISPR modifications of *let-60* and *ced-10*

The LET-60 amino acid Cys118 (C118) and the CED-10 amino acid Cys18 (C18) were modified using the CRISPR/Cas9 gene editing protocol as described [58]. Briefly, http://crispr.mit.edu was used to select crRNA sequences targeting *let-60* or *ced-10*. The *let-60* gene-specific crRNA (5’-AGGTTCCTATGGTCTTGGTAGUUUUAGAGCUAUGCUGUUUUG-3’), the *ced-10* gene-specific crRNA (5’-CGTTTGTGGTGTAGGATATCGUUUUAGAGCUAUGCUGUUUUG-3‘), the coCRISPR *dpy-10* crRNA and the tracRNA (Dharmacon) were re-suspended in 10mM Tris pH 7.4 to 8 μg/μl. The purified Cas9 protein (PNA Bio. Inc.) was reconstituted in water to 2 mg/ml. The repair templates for *let-60-C118S* (5’-TGAAATTATCAGTCAATGGTTGAATATTTGTATTTCTTCTAGGTTCCTATGGTGTTGGTAGGCAATAAATCTGATTTGTCATCTCGATCAGTCGACTTCCGAACAGTCAGTGAGACA-3’), *let-60-C118D* (5’-TGAAATTATCAGTCAATGGTTGAATATTTGTATTTCTTCTAGGTTCCTATGGTGTTGGTAGGCAATAAAGATGATTTGTCATCTCGATCAGTCGACTTCCGAACAGTCAGTGAGACA-3’) and *ced-10-C18S* (5’-ATGCAAGCGATCAAATGTGTCGTCGTTGGTGACGGAGCCGTCGGTAAAACGTCTCTACTGATCTCCTACACCACAAACGCATTTCCCGGAGAATATATTCCGACGGTGAGTCATTT-3’) (IDT) were resuspended in water to 1 μg/μl and 500 ng/μl for *dpy-10*. The injection mix contained 6.25 μl Cas9 protein, 1.25 μl tracrRNA, 0.2 μl crRNA *dpy-10*, 0.275 μl ssODN *dpy-10*, 0.5 μl crRNA *let-60* or *ced-10*, 1.1 μl ssODN *let-60-C118S*, *let-60-C118D* or *ced-10-C18S*, 0.25 μl KCl (1M) and 0.375 μl Hepes pH 7.4 (200 mM). The mix was activated for 15 min at 37°C and injected into distal gonads of young adult worms. F1 roller worms were singled out and after they laid eggs, were genotyped by single worm lysis and PCR using the primers 5’-GTGAGACATGCCTCCTCGAC-3‘ and 5‘-GGTGTCGTATTTTTGGCGCGA-3‘ for the *let-60* modifications and the primers 5’-CGTCTTGATGCCCGTTGTG-3‘ and 5‘-GCTGTATCCCAGAGCCCGA-3‘ for the *ced-10* modification. The *let-60* modifications were confirmed by sequencing the PCR product, whereas the *ced-10* modification was confirmed by digesting the PCR product with EcoRV (R0195S, New England Biolabs) since isoleucine 21 of CED-10, which is encoded by ATA codon, creates an EcoRV cut site which is removed by successful editing. The non-roller F2 worms were sequenced to verify successful editing.

### Western blotting

200 synchronized young adult worms were harvested and washed in M9 buffer, and finally placed into 50 μl of 1x SDS sample buffer. The samples were lysed at 95°C for 5 min followed by centrifugation at 13,000 rpm for 1 min. For each blot and each sample 15 μl of the supernatant was loaded on to a 10% SDS‐PAGE gel followed by electro-transfer to nitrocellulose membrane. Primary antibodies were anti-di-phosphorylated ERK-1&2 antibody (1:1000, Sigma), anti-ERK1/2 antibody (1:1000, Cell Signalling Technology), Anti-Ras antibody [EPR3255] (1:1000, Abcam), anti-Tubulin antibody (1:1000, Sigma), and anti-Histone H3 antibody (1:2000, Abcam). After overnight incubation with the primary antibodies, membranes were washed 3 times with PBS-Tween and incubated with secondary antibodies which were anti-rabbit or anti-mouse conjugated to HRP (1:2000, Cell Signalling Technology). Blots were visualized using ECL (GE Healthcare) and film. Band intensity quantification was performed using Image J software (http://imagej.nih.gov/ij/).

### Quantitative Real-Time PCR

Synchronized worms were harvested at the young-adult stage, as for Western Blotting. RNA was isolated using TRIzol reagent (Invitrogen) and was transcribed into cDNA using qScript XLT cDNA SuperMix (Quanta Biosciences). Real-time PCR was performed on a CFX96 qPCR system (Bio-Rad) with the Luna Universal Probe qPCR Master Mix (NEB) according to the manufacturer's instruction. The following primers are used: LET-60 forward: 5’- GTCAGTGAGACAGCAAAGGGT-3’, reverse: 5’-CGTGACGCTCACGATGCTTG-3’; PMP-3 forward: 5’-GTTCCCGTGTTCATCACTCAT-3’, reverse: 5’-ACACCGTCGAGAAGCTGTAGA-3’. The gene *pmp-3* was used as the normalisation control.

### Multivulva phenotype scoring

Adult worms were transferred to control NGM plates or plates containing PQ or NAC for a 3- hour limited egg-laying. Once hatched worms reached adulthood with completed vulval formation, animals were scored for the presence of a normal vulva as well as the ectopic pseudovulvas. We report the total number of vulvas, which includes both. The controls shown are always scored in parallel for every experiment. All numerical data are presented in **S1 Table**.

### Statistical analyses

For scoring of the multivulva phenotype, experimental strains were compared to the control using one-way ANOVA followed by Dunnett’s multiple comparison test, which corrects for multiple comparisons. P-values and samples sizes are reported in **S1 Table**. For comparing % Muv, the Chi-square test was used. For Western Blot quantifications, ratios of phosphorylated pMPK-1a to total MPK-1 or LET-60 levels normalised to the wild-type control were compared using one-way ANOVA followed by Dunnett’s multiple comparison test. All statistical analyses were carried out using Graphpad Prism 7.03.

## Supporting information

S1 Fig Alignment of RAS proteins from *C*. *elegans* and *D*. *melanogaster* with isoforms from humans. The amino acid affected by the *C*. *elegans n1046gf* mutation (G13) is outlined in red, and the redox-sensitive cysteine we have modified by CRISPR in this study (C118) is outlined in blue.
(TIF)

S2 Fig Effect of PQ and NAC on the Muv phenotype of three *let-60gf* alleles. Both the *n1046* and *n1700* mutation harbor the G13E mutation but were isolated independently. The *ga89* mutation is an L19F substitution that is a temperature-sensitive *gf* mutation. **A** Data is graphed as % Muv to allow for better visualisation of the effects of NAC and PQ on the weak Muv phenotype of *ga89*, even at the restrictive temperature. Data for *let-60(n1046)* comes from Fig 1D. **B** Same data as in A but graphed as Average # of Vulvas. ***P = 0.0001 and **P = 0.005 compared to control as indicated.
(TIF)

S3 Fig Oxidation of C118 of LET-60ras inhibits the specification of vulval cell fates. **A** Quantification of invaginations at the Pn.pxx stage. **B.** Representative images. Asterisks indicate the invagination that will develop into the main vulva and arrowheads point to invaginations that will lead to the development of pseudovulvas.
(TIF)

S4 Fig Western blot analysis of MPK-1 levels in WT and *let-60* mutants. **A, B** Relative expression levels of total MPK-1. Values are shown as a fraction of the ratio of the indicated proteins compared to wild-type worms. **A** Relative expression levels of total MPK-1a and MPK-1b relative to the loading control Tubulin. **B** Relative expression levels of total MPK-1a and MPK-1b relative to the loading control Histone. Mean and standard error of the mean (SEM) of 3 independent experiments are indicated in the graphs. No significant differences were detected, illustrating that the significant difference shown in Fig 3A and 3B arise from differences in the levels of pMPK-1a not total MPK-1a. **C** Original scans of western blots. 1: wild-type N2; 2:*let-60(+)*-C118S; 3:*let-60(+)*-C118D;4:*let-60gf*; 5:*let-60gf*-C118S; 6:*let-60gf*-C118D. The scanned images were cropped to improve clarity and focus upon the specific proteins. Molecular weight markers are indicated.
(TIF)

S5 Fig Western blot analysis of MPK-1 levels in WT and *let-60* mutants treated with PQ and NAC. **A** Relative expression levels of total MPK-1a to Histone H3. Mean and standard error of the mean (SEM) of 3 independent experiments are indicated in the graphs. No significant differences were detected illustrating that the significant difference shown in Fig 3C and 3D arise from differences in the levels of pMPK-1a not total MPK-1a. **B** Relative expression levels of total MPK-1b and pMPK-1b to Histone H3. Mean and standard error of the mean (SEM) of 3 independent experiments are indicated in the graph. Although PQ may affect total MPK-1b and pMPK-1b levels, due to the high degree of variability no statistically significant differences were found. **C** Original scans of western blots. The scanned images were cropped to improve clarity and focus upon the specific proteins. Molecular weight markers are indicated.
(TIF)

S6 Fig Western blot analysis of LET-60 levels in WT and *let-60* mutants. **A** Original scans of western blots for Fig 3E and 3F. A representative blot was shown in Fig 3E. Samples loaded in lanes are as follows: 1: wild-type N2; 2:*let-60*-C118S; 3:*let-60*-C118D; 4:*let-60gf*; 5:*let-60gf*-C118S; 6:*let-60gf*-C118D. **B** Original scans of western blots for Fig 3G and 3H. A representative blot was shown in Fig 3G. Samples loaded in lanes are as follows: 1: wild-type N2; 2: WT + PQ; 3: WT + NAC; 4:*let-60gf*; 5:*let-60gf* + PQ; 6:*let-60gf* + NAC. The scanned images were cropped to improve clarity and focus upon the specific proteins. Molecular weight markers are indicated.
(TIF)

S7 Fig mRNA expression levels of *let-60ras* in C118S and C11D mutants. Comparison of expression levels of *let-60ras* gene in wild-type N2; *let-60gf; let-60(+)*-C118D; *let-60gf*-C118D; *let-60(+)*-C118S; and *let-60gf*-C118S backgrounds. Expression levels are normalised to the wild-type. Results represent the average of three independent biological samples, each of which was amplified two times in triplicate.
(TIF)

S8 Fig Western blot analysis of MPK-1 levels in *isp-1* and *nuo-6* mutants. **A** Relative expression levels of total MPK-1a relative to the loading control histone H3. Values are shown as a fraction of the ratio of the indicated proteins compared to wild-type worms. No significant differences were detected, illustrating that the significant differences shown in Fig 3G and 3F arise from differences in the levels of pMPK-1a not total MPK-1a. **B** Relative expression levels of total MPK-1b and pMPK-1b relative to the loading control histone H3. MPK-1b level was significantly decreased in *nuo-6* and *nuo-6; let-60gf* compared to wild-type. It also appeared to be decreased in *isp-1* and *let-60gf*, although these differences were not statistically significant. The changes of dpMPK-1b levels mirrors the changes of total MPK-1b but the decrease of dpMPK-1b levels in *isp-1* and *let-60gf* reached statistical significance. Mean and standard error of the mean (SEM) of 3 independent experiments are indicated in the graphs. **C** Original scans of western blots. 1: wild-type N2; 2:*nuo-6*; 3:*isp-1*; 4: *let-60gf*; 5:nuo-6; *let-60gf*; 6: *isp-1 et-60gf*.The scanned images were cropped to improve clarity and focus upon the specific proteins. Molecular weight markers are indicated.
(TIF)

S9 Fig Alignment of Rac proteins from *C*. *elegans*, *D*. *melanogaster* and humans. The redox-sensitive cysteine we have modified by CRISPR in this study (C18) is outlined in blue.
(TIF)

S1 Table Numerical values for Muv data shown in bar graphs in all figures. (DOCX)
