# Supplementary figures and images for: ROS regulation of RAS and vulva development in Caenorhabditis elegans
Source: PLoS Genet. 2020 Jun 16;16(6):e1008838. doi: 10.1371/journal.pgen.1008838 (PMC7319342; doi:10.1371/journal.pgen.1008838)

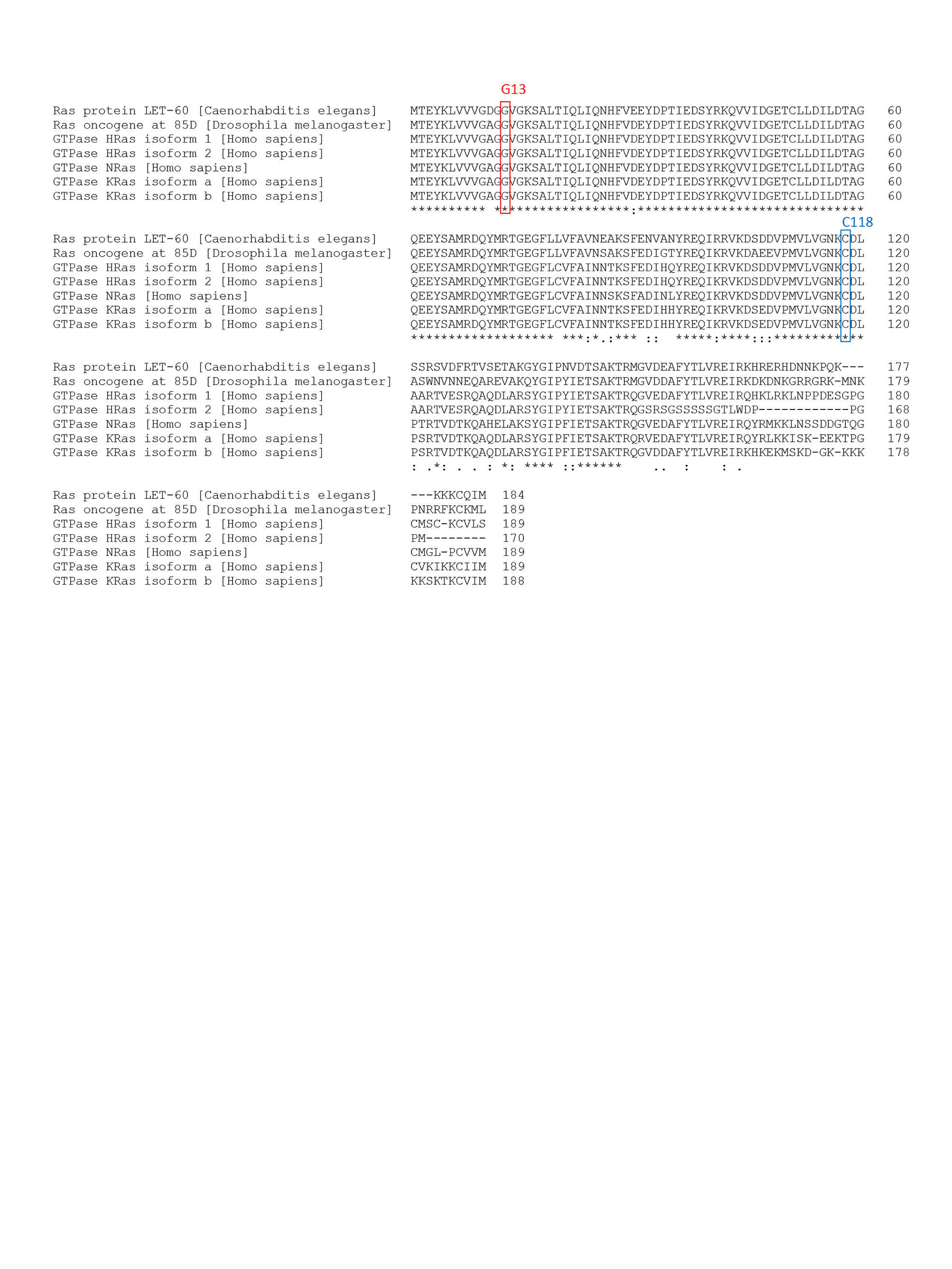

Supplement: S1 Fig — The amino acid affected by the C. elegans n1046gf mutation (G13) is outlined in red, and the redox-sensitive cysteine we have modified by CRISPR in this study (C118) is outlined in blue. (TIF) [file pgen.1008838.s001.tif]

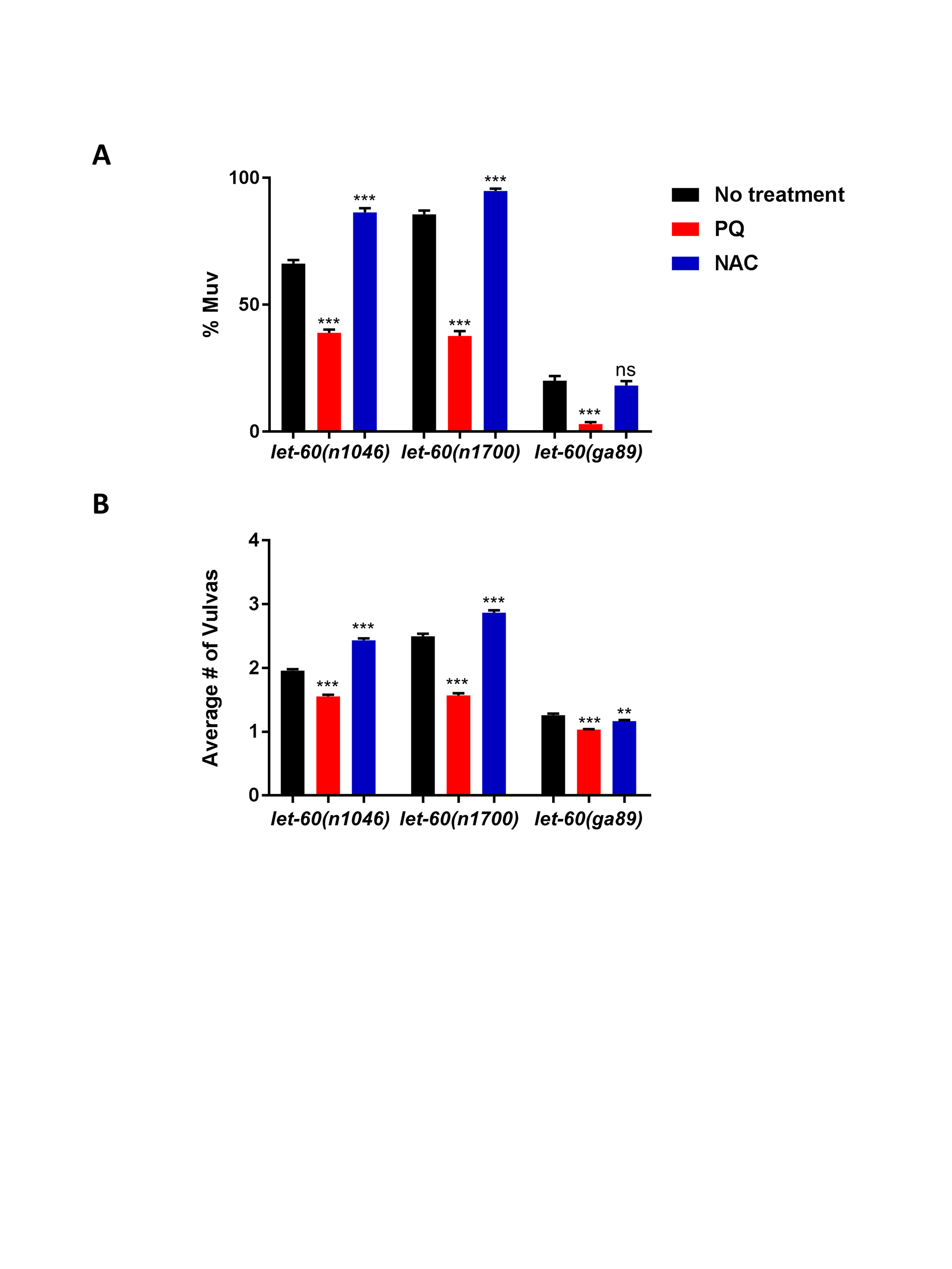

Supplement: S2 Fig — Both the n1046 and n1700 mutation harbor the G13E mutation but were isolated independently. The ga89 mutation is an L19F substitution that is a temperature-sensitive gf mutation. A Data is graphed as % Muv to allow for better visualisation of the effects of NAC and PQ on the weak Muv phenotype of ga89, even at the restrictive temperature. Data for let-60(n1046) comes from Fig 1D. B Same data as in A but graphed as Average # of Vulvas. ***P = 0.0001 and **P = 0.005 compared to control as indicated. (TIF) [file pgen.1008838.s002.tif]

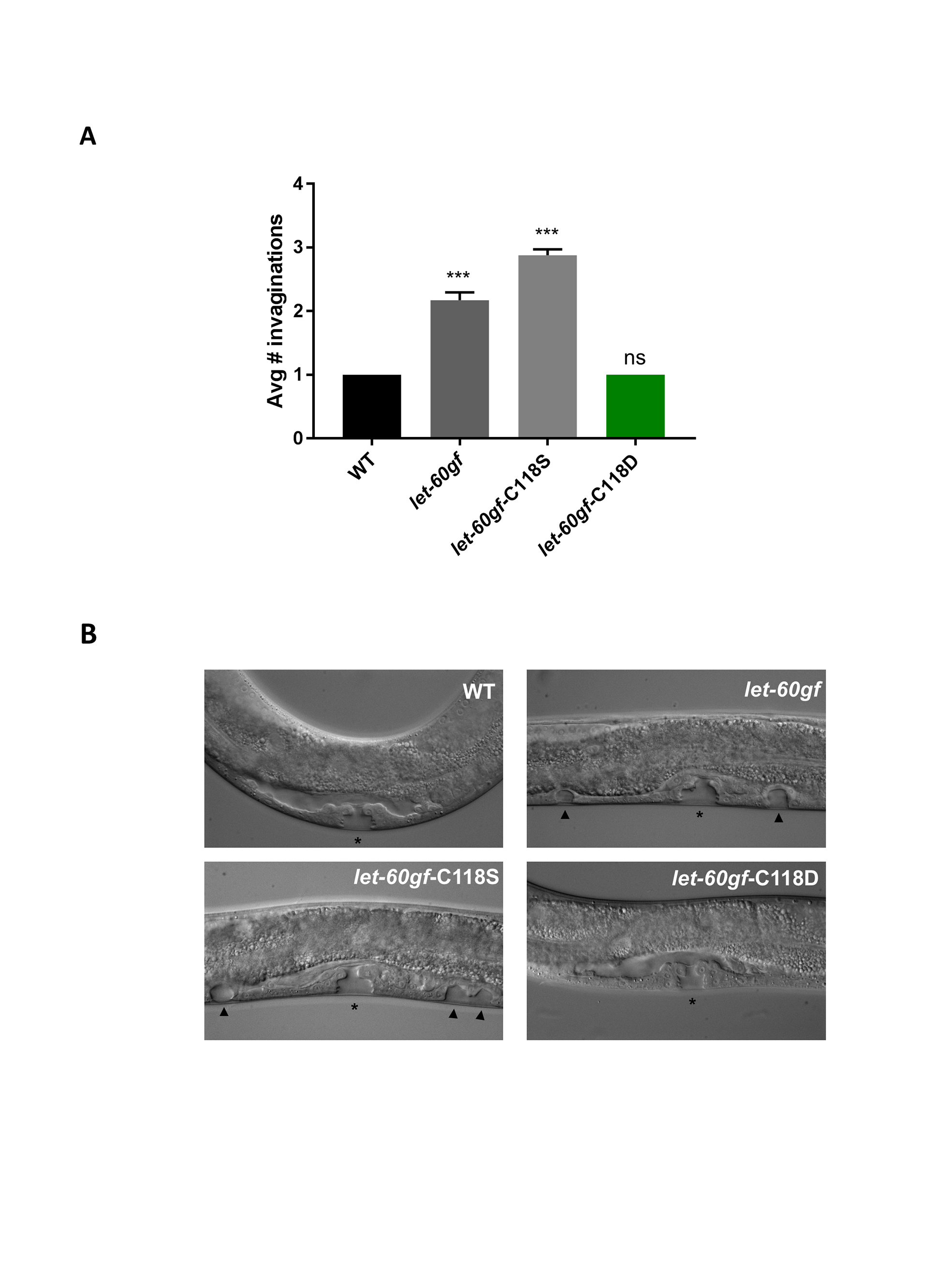

Supplement: S3 Fig — A Quantification of invaginations at the Pn.pxx stage. B. Representative images. Asterisks indicate the invagination that will develop into the main vulva and arrowheads point to invaginations that will lead to the development of pseudovulvas. (TIF) [file pgen.1008838.s003.tif]

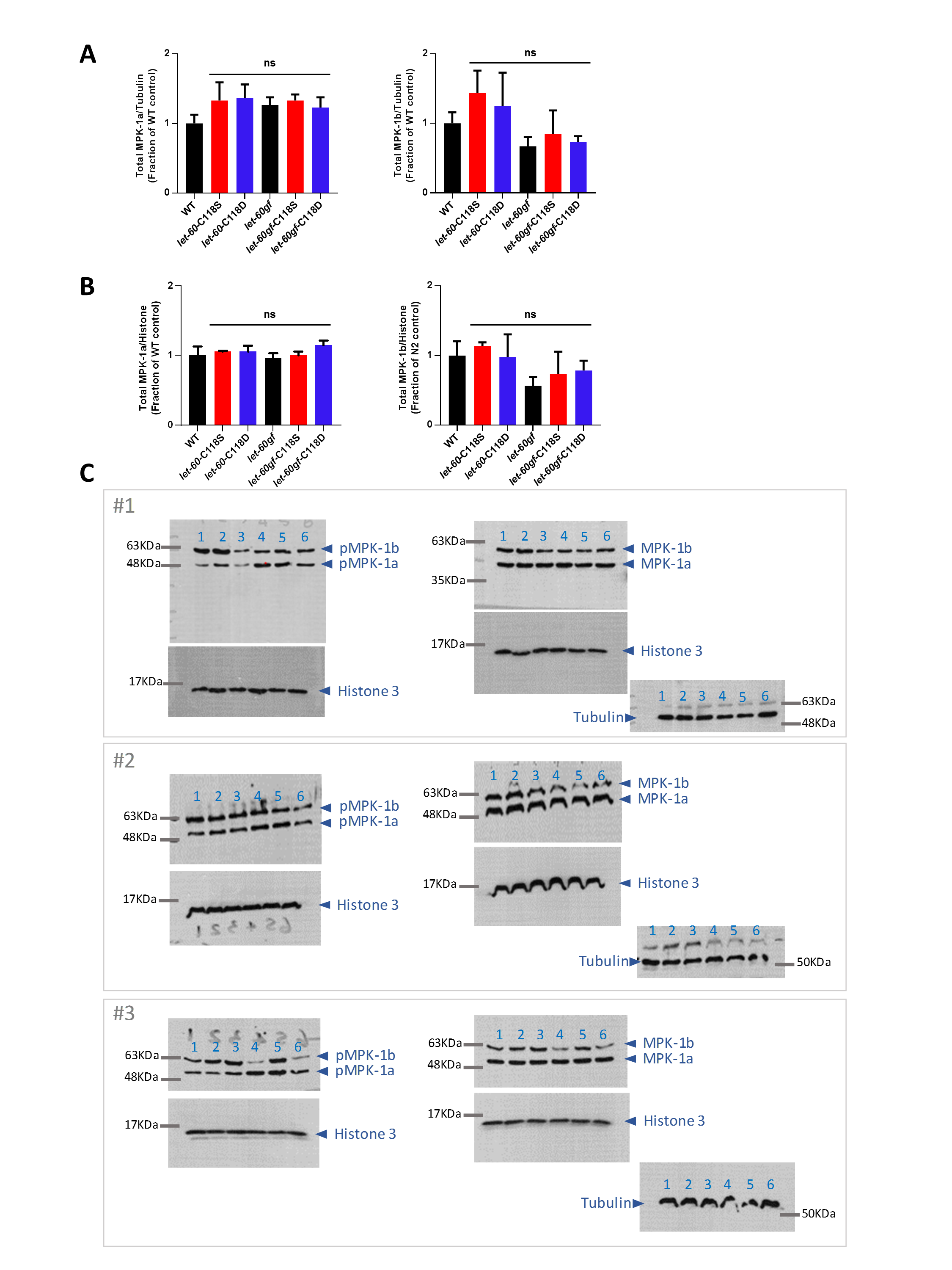

Supplement: S4 Fig — A, B Relative expression levels of total MPK-1. Values are shown as a fraction of the ratio of the indicated proteins compared to wild-type worms. A Relative expression levels of total MPK-1a and MPK-1b relative to the loading control Tubulin. B Relative expression levels of total MPK-1a and MPK-1b relative to the loading control Histone. Mean and standard error of the mean (SEM) of 3 independent experiments are indicated in the graphs. No significant differences were detected, illustrating that the significant difference shown in Fig 3A and 3B arise from differences in the levels of pMPK-1a not total MPK-1a. C Original scans of western blots. 1: wild-type N2; 2:let-60(+)-C118S; 3:let-60(+)-C118D;4:let-60gf; 5:let-60gf-C118S; 6:let-60gf-C118D. The scanned images were cropped to improve clarity and focus upon the specific proteins. Molecular weight markers are indicated. (TIF) [file pgen.1008838.s004.tif]

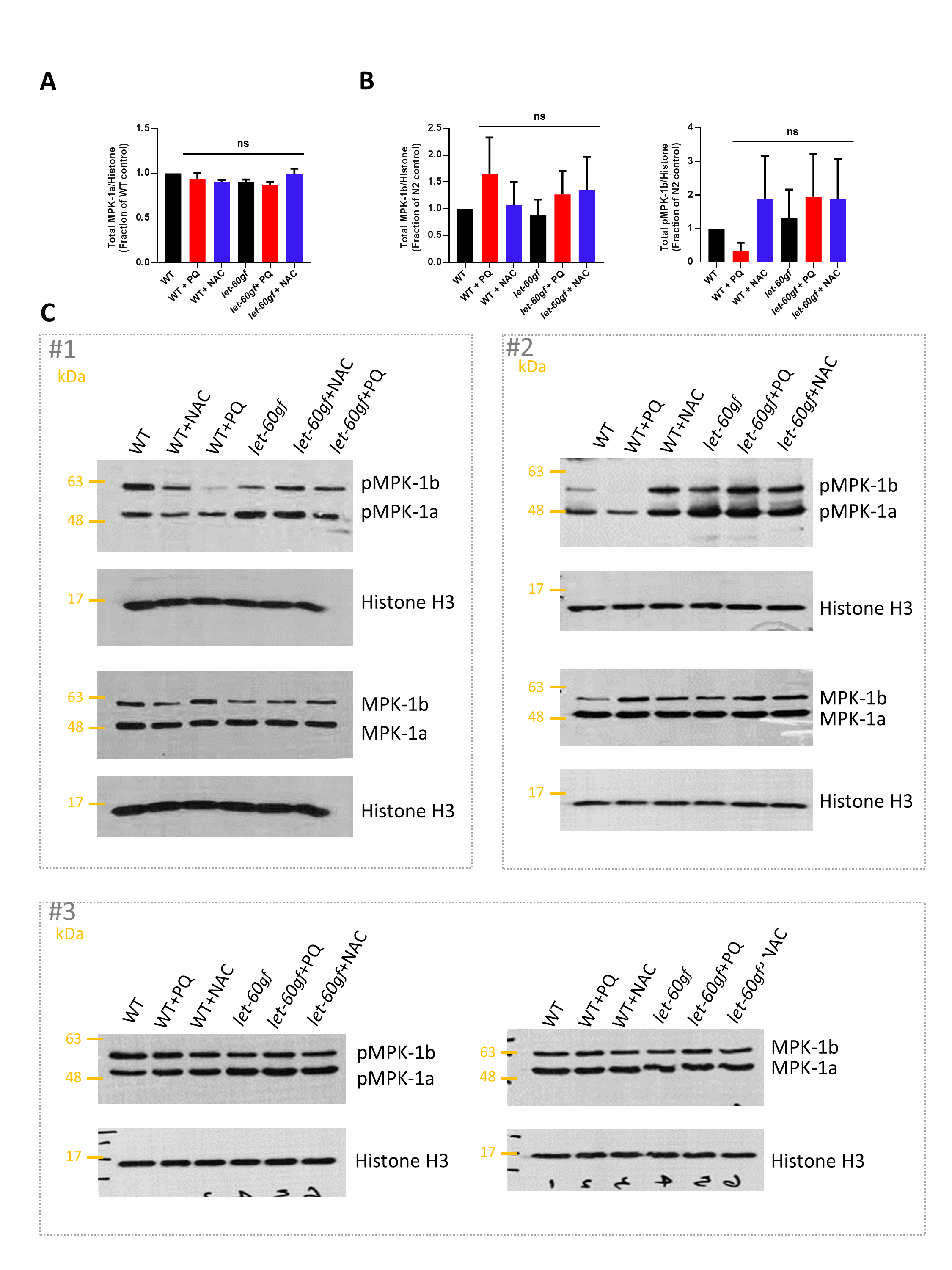

Supplement: S5 Fig — A Relative expression levels of total MPK-1a to Histone H3. Mean and standard error of the mean (SEM) of 3 independent experiments are indicated in the graphs. No significant differences were detected illustrating that the significant difference shown in Fig 3C and 3D arise from differences in the levels of pMPK-1a not total MPK-1a. B Relative expression levels of total MPK-1b and pMPK-1b to Histone H3. Mean and standard error of the mean (SEM) of 3 independent experiments are indicated in the graph. Although PQ may affect total MPK-1b and pMPK-1b levels, due to the high degree of variability no statistically significant differences were found. C Original scans of western blots. The scanned images were cropped to improve clarity and focus upon the specific proteins. Molecular weight markers are indicated. (TIF) [file pgen.1008838.s005.tif]

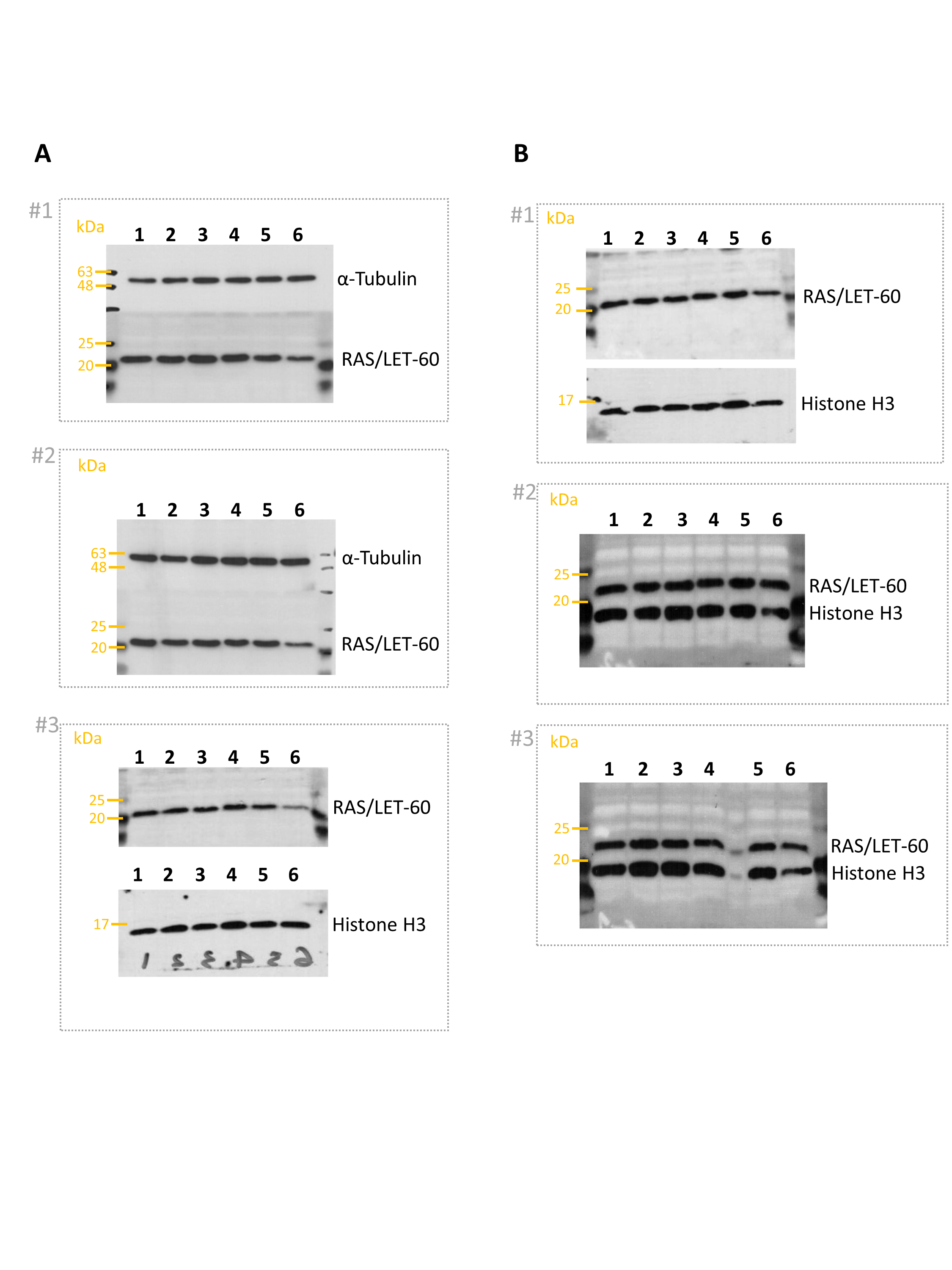

Supplement: S6 Fig — A Original scans of western blots for Fig 3E and 3F. A representative blot was shown in Fig 3E. Samples loaded in lanes are as follows: 1: wild-type N2; 2:let-60-C118S; 3:let-60-C118D; 4:let-60gf; 5:let-60gf-C118S; 6:let-60gf-C118D. B Original scans of western blots for Fig 3G and 3H. A representative blot was shown in Fig 3G. Samples loaded in lanes are as follows: 1: wild-type N2; 2: WT + PQ; 3: WT + NAC; 4:let-60gf; 5:let-60gf + PQ; 6:let-60gf + NAC. The scanned images were cropped to improve clarity and focus upon the specific proteins. Molecular weight markers are indicated. (TIF) [file pgen.1008838.s006.tif]

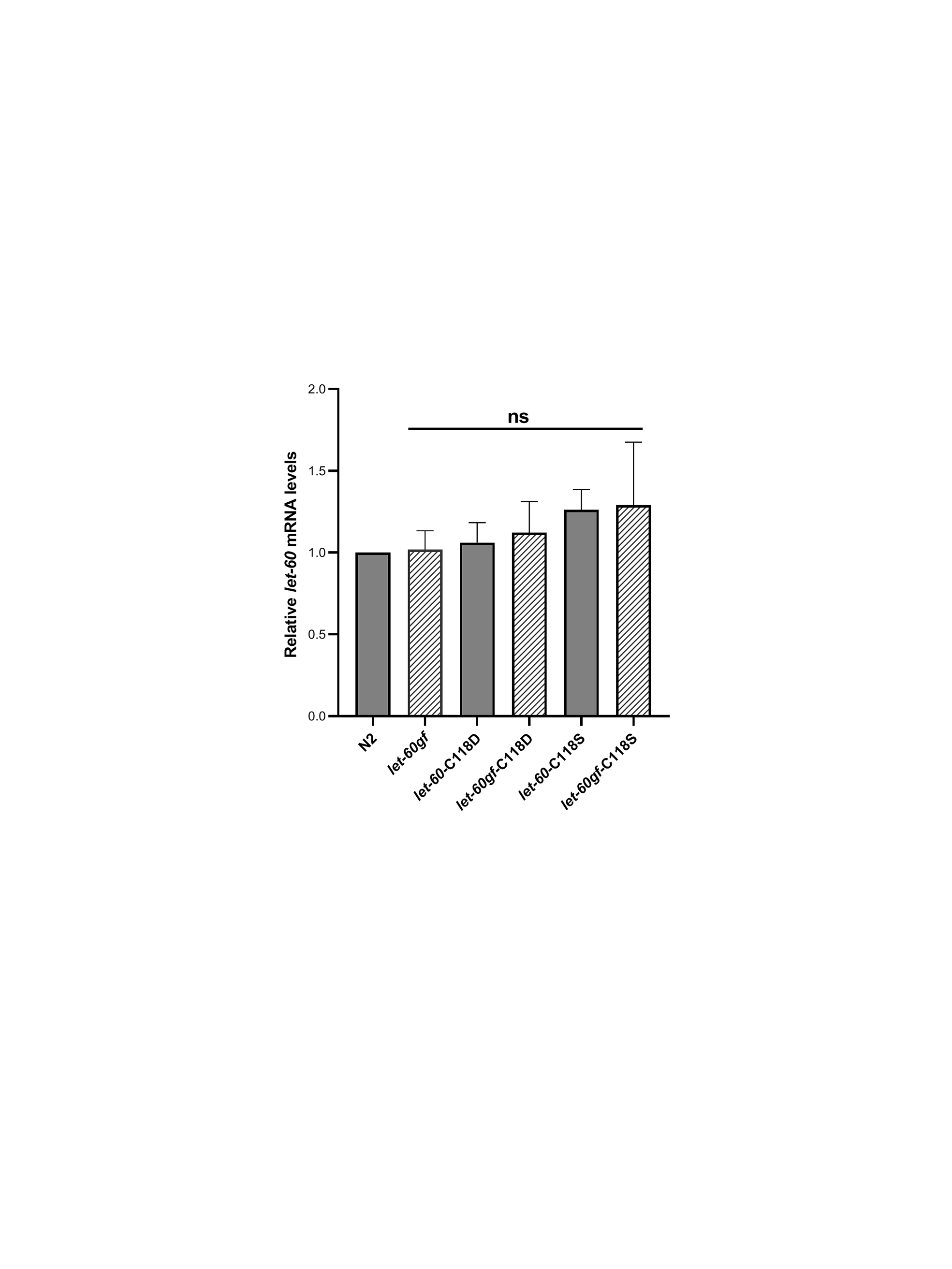

Supplement: S7 Fig — Comparison of expression levels of let-60ras gene in wild-type N2; let-60gf; let-60(+)-C118D; let-60gf-C118D; let-60(+)-C118S; and let-60gf-C118S backgrounds. Expression levels are normalised to the wild-type. Results represent the average of three independent biological samples, each of which was amplified two times in triplicate. (TIF) [file pgen.1008838.s007.tif]

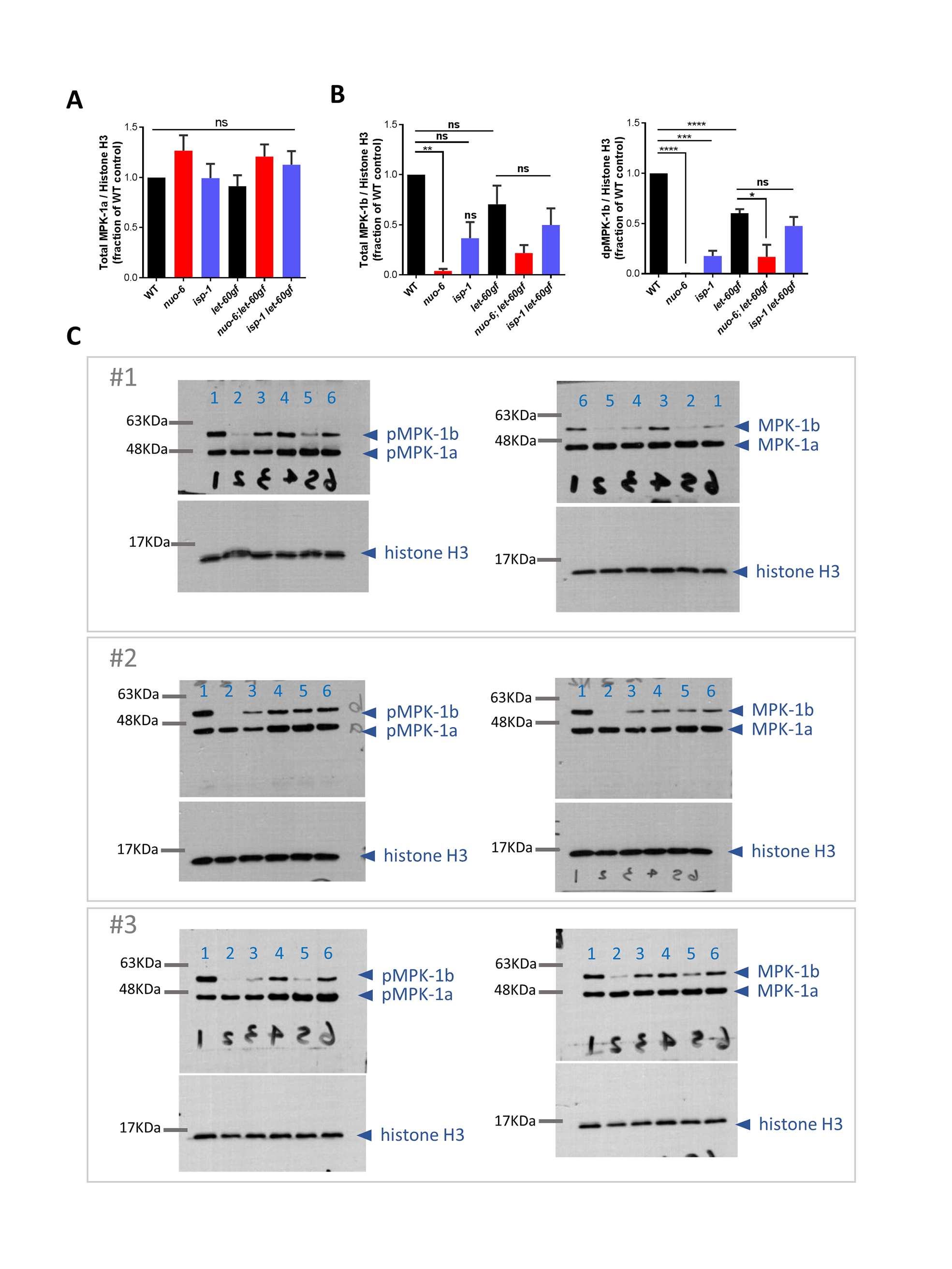

Supplement: S8 Fig — A Relative expression levels of total MPK-1a relative to the loading control histone H3. Values are shown as a fraction of the ratio of the indicated proteins compared to wild-type worms. No significant differences were detected, illustrating that the significant differences shown in Fig 3G and 3F arise from differences in the levels of pMPK-1a not total MPK-1a. B Relative expression levels of total MPK-1b and pMPK-1b relative to the loading control histone H3. MPK-1b level was significantly decreased in nuo-6 and nuo-6; let-60gf compared to wild-type. It also appeared to be decreased in isp-1 and let-60gf, although these differences were not statistically significant. The changes of dpMPK-1b levels mirrors the changes of total MPK-1b but the decrease of dpMPK-1b levels in isp-1 and let-60gf reached statistical significance. Mean and standard error of the mean (SEM) of 3 independent experiments are indicated in the graphs. C Original scans of western blots. 1: wild-type N2; 2:nuo-6; 3:isp-1; 4: let-60gf; 5:nuo-6; let-60gf; 6: isp-1 et-60gf.The scanned images were cropped to improve clarity and focus upon the specific proteins. Molecular weight markers are indicated. (TIF) [file pgen.1008838.s008.tif]

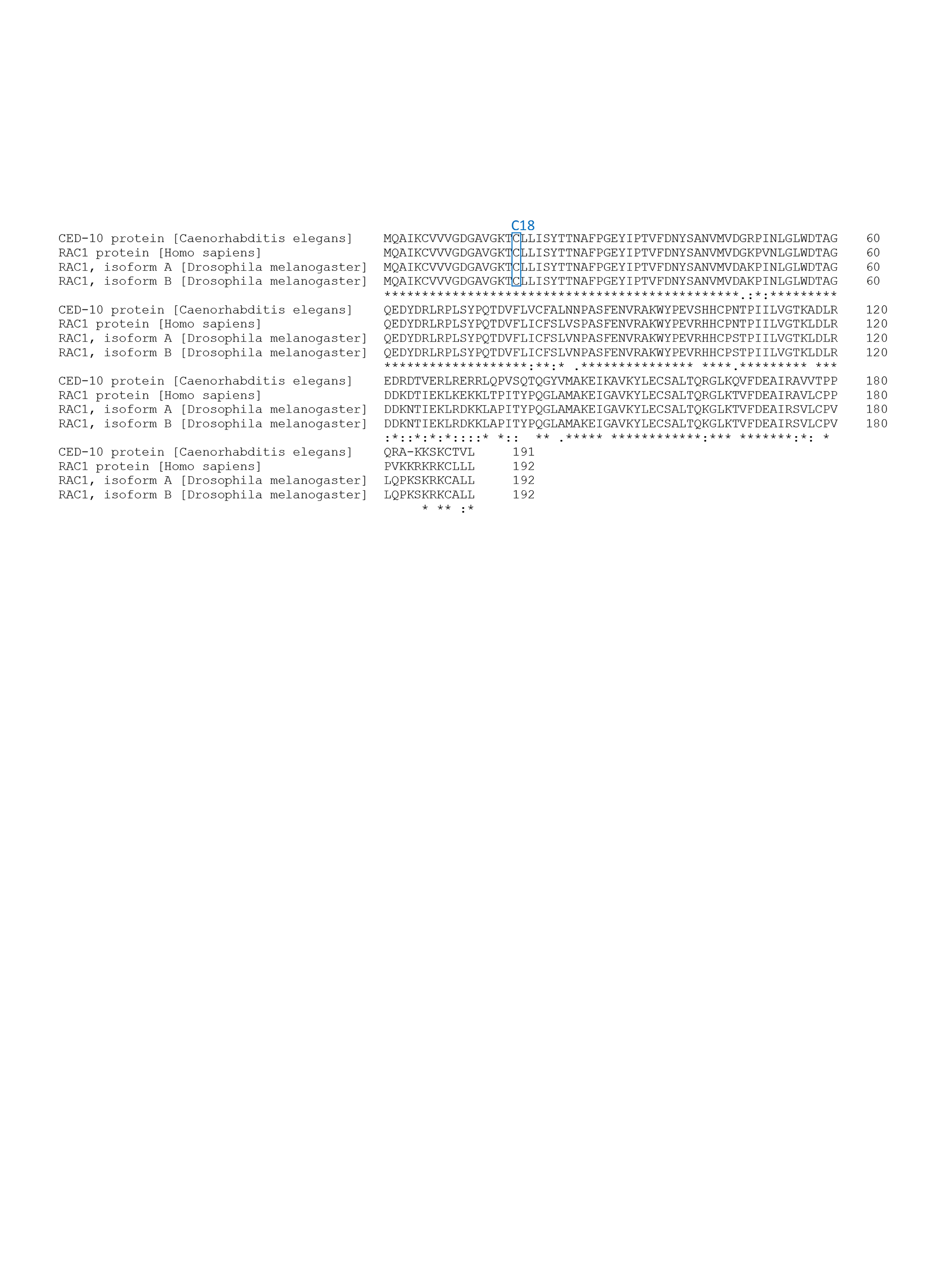

Supplement: S9 Fig — The redox-sensitive cysteine we have modified by CRISPR in this study (C18) is outlined in blue. (TIF) [file pgen.1008838.s009.tif]
