# Supplementary material for: ROS regulation of RAS and vulva development in Caenorhabditis elegans
Source: PLoS Genet. 2020 Jun 16;16(6):e1008838. doi: 10.1371/journal.pgen.1008838 (PMC7319342; doi:10.1371/journal.pgen.1008838)
Supplement: S1 Table — (DOCX) [file pgen.1008838.s010.docx]

**Supporting Table 1.** Numerical values and statistics for Muv data Presented in Figures 1-5 and Supplemental Figure 2.

| Figure Panel | Genotype | Sample size | Mean # of vulva  ± SEM | Adjusted  P value^1,2^ |
| --- | --- | --- | --- | --- |
|  |  |  |  |  |
| **1D** | *let-60rasgf* | 1091 | 1.958 ± 0.02521 |  |
|  | *let-60rasgf* +0.1mM PQ | 857 | 1.554 ± 0.02707 | 0.0001 |
|  | *let-60rasgf* +9mM NAC | 780 | 2.436 ± 0.02914 | 0.0001 |
|  |  |  |  |  |
| **2D** | *let-60rasgf* | 1733 | 2.081 ± 0.02062 |  |
|  | *let-60rasgf*+0.1mM PQ | 404 | 1.51 ± 0.03942 | 0.0001 |
|  | *let-60rasgf*+9mM NAC | 1295 | 2.484 ± 0.02324 | 0.0001 |
|  | *let-60rasgf*-C118S | 1326 | 2.604 ± 0.02418 | 0.0001 |
|  | *let-60rasgf*-C118S +0.1mM PQ | 380 | 2.597 ± 0.05292 | 0.0001 |
|  | *let-60rasgf*-C118S +9mM NAC | 1352 | 2.632 ± 0.02274 | 0.0001 |
| **2D** |  |  |  |  |
|  | *let-60rasgf*-C118S | 1326 | 2.604 ± 0.02418 |  |
|  | *let-60rasgf*-C118S +0.1mM PQ | 380 | 2.597 ± 0.05292 | 0.9875 |
|  | *let-60rasgf*-C118S +9mM NAC | 1352 | 2.632 ± 0.02274 | 0.6161 |
|  |  |  |  |  |
| **2E** | *let-60rasgf* | 753 | 1.903 ± 0.02838 |  |
|  | *let-60rasgf*+0.1mM PQ | 450 | 1.658 ± 0.03771 | 0.0001 |
|  | *let-60rasgf*+9mM NAC | 460 | 2.32± 0.03881 | 0.0001 |
|  | *let-60rasgf*-C118D | 751 | 1.0 ± 0.00 | 0.0001 |
|  | *let-60rasgf*-C118D +0.1mM PQ | 450 | 1.0 ± 0.00 | 0.0001 |
|  | *let-60rasgf*-C118D +9mM NAC | 450 | 1.0 ± 0.00 | 0.0001 |
|  |  |  |  |  |
| **3A** | *let-60rasgf* | 624 | 1.970 ± 0.03296 |  |
|  | *let-60rasgf*+0.1mM PQ | 453 | 1.594 ± 0.03719 | 0.0001 |
|  | *let-60rasgf*+9mM NAC | 454 | 2.474 ± 0.03852 | 0.0001 |
|  | *sod-1; let-60rasgf* | 608 | 2.933 ± 0.03107 | 0.0001 |
|  | *sod-1; let-60rasgf* +0.1mM PQ | 485 | 3.324 ± 0.03971 | 0.0001 |
|  | *sod-1; let-60rasgf* +9mM NAC | 428 | 3.037 ± 0.03811 | 0.0001 |
|  | *sod-1; let-60rasgf*-C118S | 930 | 3.002 ± 0.02509 | 0.0001 |
|  | *sod-1; let-60rasgf*-C118S +0.1mM PQ | 380 | 3.842 ± 0.03787 | 0.0001 |
|  | *sod-1; let-60rasgf*-C118S +9mM NAC | 406 | 3.01 ± 0.03723 | 0.0001 |
|  |  |  |  |  |
| **3A** | *sod-1; let-60rasgf* | 608 | 2.933 ± 0.03107 |  |
|  | *sod-1; let-60rasgf* +0.1mM PQ | 485 | 3.324 ± 0.03971 | 0.0001 |
|  | *sod-1; let-60rasgf* +9mM NAC | 428 | 3.037 ± 0.03811 | 0.0733 |
|  |  |  |  |  |
| **3A** | *sod-1; let-60rasgf*-C118S | 629 | 2.949 ± 0.03111 |  |
|  | *sod-1; let-60rasgf*-C118S +0.1mM PQ | 405 | 3.906 ± 0.04642 | 0.0001 |
|  | *sod-1; let-60rasgf*-C118S +9mM NAC | 426 | 3.01 ± 0.03723 | 0.3490 |
|  |  |  |  |  |
| **3A** | *let-60rasgf*+0.1mM PQ | 453 | 1.594 ± 0.03719 |  |
|  | *sod-1; let-60rasgf* +0.1mM PQ | 485 | 3.324 ± 0.03971 | 0.0001 |
|  | *sod-1; let-60rasgf*-C118S +0.1mM PQ | 405 | 3.906 ± 0.04642 | 0.0001 |
|  |  |  |  |  |
| **3A** | *let-60rasgf*+9mM NAC | 454 | 2.474 ± 0.03852 |  |
|  | *sod-1; let-60rasgf* +9mM NAC | 428 | 3.037 ± 0.03811 | 0.0001 |
|  | *sod-1; let-60rasgf*-C118S +9mM NAC | 426 | 3.01 ± 0.03723 | 0.0001 |
|  |  |  |  |  |
| **3A** | *sod-1; let-60rasgf* +0.1mM PQ | 485 | 3.324 ± 0.03971 |  |
|  | *sod-1; let-60rasgf*-C118S +0.1mM PQ | 405 | 3.906 ± 0.04642 | 0.0012 |
|  |  |  |  |  |
| **3A** | *sod-1; let-60rasgf* +9mM NAC | 428 | 3.037 ± 0.03811 |  |
|  | *sod-1; let-60rasgf*-C118S +9mM NAC | 426 | 3.01 ± 0.03723 | 0.6059 |
|  |  |  |  |  |
| **3B** | *let-60rasgf* | 878 | 1.948 ± 0.03820 |  |
|  | *nuo-6; let-60rasgf* | 631 | 1.087 ± 0.01231 | 0.0001 |
|  | *isp-1 let-60rasgf* | 458 | 1.048 ± 0.01000 | 0.0001 |
|  | *sod-2; let-60rasgf* | 610 | 1.526 ± 0.03026 | 0.0001 |
|  |  |  |  |  |
| **3B** | *let-60rasgf*-C118S | 600 | 2.587 ± 0.03457 |  |
|  | *isp-1 let-60rasgf-*C118S | 459 | 1.749 ± 0.04011 | 0.0001 |
|  | *nuo-6; let-60rasgf-*C118S | 453 | 1.404 ± 0.03286 | 0.0001 |
|  | *sod-2; let-60rasgf*-C118S | 462 | 1.978 ± 0.04099 | 0.0001 |
|  |  |  |  |  |
| **3B** | *let-60rasgf* | 878 | 1.948 ± 0.03820 |  |
|  | *let-60rasgf*-C118S | 600 | 2.587 ± 0.03457 | 0.0001 |
|  |  |  |  |  |
| **3B** | *isp-1 let-60rasgf* | 458 | 1.048 ± 0.01000 |  |
|  | *isp-1 let-60rasgf-*C118S | 459 | 1.749 ± 0.04011 | 0.0001 |
|  |  |  |  |  |
| **3B** | *nuo-6; let-60rasgf* | 631 | 1.087 ± 0.01231 |  |
|  | *nuo-6; let-60rasgf-*C118S | 453 | 1.404 ± 0.03286 | 0.0001 |
|  |  |  |  |  |
| **3B** | *sod-2; let-60rasgf* | 610 | 1.526 ± 0.03026 |  |
|  | *sod-2; let-60rasgf*-C118S | 462 | 1.978 ± 0.04099 | 0.0001 |
|  |  |  |  |  |
| **3C** | *let-60rasgf* | 307 | 1.870 ± 0.04507 |  |
|  | *sod-1; let-60rasgf* | 428 | 2.593 ± 0.04283 | 0.0001 |
|  | *sod-2; let-60rasgf* | 423 | 1.487 ± 0.03311 | 0.0001 |
|  | *sod-1; sod-2; let-60rasgf* | 468 | 2.244 ± 0.03969 | 0.0001 |
|  |  |  |  |  |
| **3C** | *sod-2; let-60rasgf* | 423 | 1.487 ± 0.03311 |  |
|  | *sod-1; sod-2; let-60rasgf* | 468 | 2.244 ± 0.03969 | 0.0001 |
|  |  |  |  |  |
| **3D** | *let-60rasgf* | 442 | 1.887 ± 0.04003 |  |
|  | *let-60rasgf* +9mM NAC | 562 | 2.375 ± 0.03579 | 0.0001 |
|  | *sod-2; let-60rasgf* | 290 | 1.555 ± 0.04472 | 0.0001 |
|  | *sod-2; let-60rasgf* +9mM NAC | 352 | 1.918 ± 0.0468 | 0.9197 |
|  |  |  |  |  |
| **3D** | *sod-2; let-60rasgf* | 290 | 1.555 ± 0.04472 |  |
|  | *sod-2; let-60rasgf* +9mM NAC | 352 | 1.918 ± 0.0468 | 0.0001 |
|  |  |  |  |  |
| **4A** | *let-60rasgf* | 837 | 1.928 ± 0.03042 |  |
|  | *sod-1; let-60rasgf* | 273 | 2.359 ± 0.052 | 0.0001 |
|  |  |  |  |  |
| **4A** | *lin-1* | 668 | 2.307 ± 0.03269 |  |
|  | *sod-1; lin-1* | 391 | 2.678 ± 0.04219 | 0.0001 |
|  |  |  |  |  |
| **4B** | *let-60rasgf* | 477 | 1.945 ± 0.04029 |  |
|  | *let-60rasgf* +0.1mM PQ | 348 | 1.652 ± 0.04354 | 0.0001 |
|  |  |  |  |  |
| **4B** | *lin-1* | 435 | 2.184 ± 0.03968 |  |
|  | *lin-1*+0.1mM PQ | 170 | 2.618 ± 0.06984 | 0.0001 |
|  |  |  |  |  |
| **4B** | *sod-1; lin-1* | 233 | 2.901 ± 0.05315 |  |
|  | *sod-1; lin-1*+0.1mM PQ | 288 | 4.094 ± 0.03689 | 0.0001 |
|  |  |  |  |  |
| **4B** | *lin-1*+0.1mM PQ | 170 | 2.618 ± 0.06984 |  |
|  | *sod-1; lin-1*+0.1mM PQ | 288 | 4.094 ± 0.03689 | 0.0001 |
|  |  |  |  |  |
| **5A** | *let-60rasgf* | 1030 | 1.930 ± 0.02550 |  |
|  | *bli-3; let-60rasgf* | 325 | 1.498 ± 0.03688 | 0.0001 |
|  | *duox-2; let-60rasgf* | 551 | 1.681 ± 0.03060 | 0.0001 |
|  |  |  |  |  |
| **5A** | *ced-10-*C18S *let-60gf* | 607 | 2.423 ± 0.03695 |  |
|  | *bli-3; ced-10-*C18S *let-60gf* | 451 | 1.769 ± 0.03108 | 0.0001 |
|  | *duox-2; ced-10-*C18S *let-60gf* | 453 | 1.731 ± 0.03488 | 0.0001 |
|  |  |  |  |  |
| **5A** | *sod-1; let-60rasgf* | 608 | 2.931 ± 0.03111 |  |
|  | *bli-3; sod-1; let-60rasgf* | 1207 | 2.334 ± 0.02101 | 0.0001 |
|  | *duox-2; sod-1; let-60rasgf* | 1209 | 2.392 ± 0.02469 | 0.0001 |
|  |  |  |  |  |
| **5A** | *let-60rasgf* | 1030 | 1.930 ± 0.02550 |  |
|  | *ced-10-*C18S *let-60gf* | 607 | 2.423 ± 0.03695 | 0.0001 |
|  |  |  |  |  |
| **5A** | *ced-10-*C18S *let-60gf* | 607 | 2.423 ± 0.03695 |  |
|  | *sod-1; let-60rasgf* | 608 | 2.931 ± 0.03111 | 0.0001 |
|  |  |  |  |  |
| **5B** | *let-60rasgf* | 927 | 1.981 ± 0.02814 |  |
|  | *let-60rasgf*+0.1mM PQ | 453 | 2.411 ± 0.04012 | 0.0001 |
|  | *let-60rasgf*+9mM NAC | 457 | 1.578 ± 0.03542 | 0.0001 |
|  |  |  |  |  |
| **5B** | *bli-3; let-60rasgf* | 907 | 1.578 ± 0.02215 |  |
|  | *bli-3; let-60rasgf* +0.1mM PQ | 375 | 1.555 ± 0.03569 | 0.8153 |
|  | *bli-3; let-60rasgf* +9mM NAC | 479 | 1.601 ± 0.03576 | 0.7789 |
|  |  |  |  |  |
| **5C** | *let-60rasgf* | 613 | 1.967 ± 0.03263 |  |
|  | *let-60rasgf*+0.1mM PQ | 326 | 2.360 ± 0.04805 | 0.0001 |
|  | *let-60rasgf*+9mM NAC | 300 | 1.472 ± 0.04136 | 0.0001 |
|  |  |  |  |  |
| **5C** | *duox-2; let-60rasgf* | 607 | 1.671 ± 0.03129 |  |
|  | *duox-2; let-60rasgf* +0.1mM PQ | 302 | 1.785 ± 0.04726 | 0.0627 |
|  | *duox-2; let-60rasgf* +9mM NAC | 302 | 1.517 ± 0.03972 | 0.0084 |
|  |  |  |  |  |
| S2B | *let-60(n1700)* | 498 | 2.498 ± 0.04082 |  |
|  | *let-60(n1700)*+0.1mM PQ | 566 | 2.869 ± 0.03591 | 0.0001 |
|  | *let-60(n1700)*+9mM NAC | 658 | 1.573 ± 0.03208 | 0.0001 |
|  |  |  |  |  |
|  |  |  |  |  |
|  | *let-60(ga89ts)* | 511 | 1.260 ± 0.02465 |  |
|  | *let-60(ga89ts)*+0.1mM PQ | 599 | 1.169 ± 0.01704 | 0.0001 |
|  | *let-60(ga89ts)*+9mM NAC | 516 | 1.035 ± 0.00809 | 0.005 |
|  |  |  |  |  |

^1^ P-values are from the one-way ANOVA test followed by the Dunnett’s multiple comparison test, which corrects for multiple comparisons.

^2^The control to which the experimental conditions are compared is given in the first row of each section of the table. Different comparisons made for the same figure panel are separated by a single line; comparisons made for different figure panels are separated by a double line.
